# Frequency-Preserving Readout Design and Error Characterization for CWT-Based rPPG Heart-Rate Estimation

**DOI:** 10.3390/s26175637

**Published:** 2026-09-04

**Authors:** Kota Toyama, Masato Takahashi, Norimichi Tsumura

**Affiliations:** 1 Graduate School of Informatics, Chiba University, 1-33 Yayoi, Inage, Chiba 263-8522, Japan; mat_diver@chiba-u.jp; 2 Hiroshima University Hospital, Hiroshima University, Hiroshima 734-8551, Japan

**Keywords:** rPPG, heart-rate estimation, wavelet transform, scalogram, soft-argmax, error characterization

## Abstract

**Highlights:**

**What are the main findings?**

- A frequency-preserving convolutional neural network with a structured soft-argmax readout significantly reduced subject-level heart-rate estimation error compared with a global average pooling readout using the same frequency-preserving backbone on PURE and UBFC-rPPG.
- Error analyses showed that major failures could originate upstream of the readout, while the evaluated continuous wavelet transform-based estimators showed smaller illumination-induced degradation than the evaluated fast Fourier transform-based estimators.

**What are the implications of the main findings?**

- For heart-rate estimation from continuous wavelet transform scalograms, a structured frequency-localizing readout provides a more reliable mapping to beats per minute than global average pooling within the evaluated architecture.
- Reliable continuous wavelet transform-based remote heart rate monitoring requires both an appropriate readout design and mechanisms for detecting or mitigating degraded and out-of-distribution input signals.

**Abstract:**

Remote photoplethysmography (rPPG) estimates heart rate (HR) from facial color variations. In continuous wavelet transform (CWT) scalograms, HR is represented by position along the frequency axis, whereas global average pooling (GAP) does not retain this coordinate explicitly. We investigated whether a structured frequency-localizing readout improves HR estimation and analyzed the remaining errors. Leave-one-subject-out evaluations were conducted on three datasets (PURE, UBFC-rPPG, and BH-rPPG) using a fixed chrominance-based signal-extraction and CWT pipeline. The proposed model combined a frequency-preserving convolutional neural network backbone with a soft-argmax readout over HR coordinates. Using the same backbone and training protocol, soft-argmax reduced segment-pooled mean absolute error (MAE) relative to the matched GAP readout from 8.09 to 2.98 beats per minute on PURE and from 5.14 to 3.82 beats per minute on UBFC-rPPG, with significant subject-level improvements on both datasets. Residual analyses indicated that major errors could originate from color projection, non-cardiac spectral components, motion, and HR-range mismatch. On BH-rPPG, the proposed model achieved the lowest MAE under low illumination, while the evaluated CWT-based estimators showed smaller illumination-induced degradation than the evaluated fast Fourier transform (FFT)-based estimators. These findings support structured soft-argmax readout design and highlight the need to detect or mitigate degraded input signals.

## 1. Introduction

Remote photoplethysmography (rPPG) estimates heart rate (HR) from subtle skin-color variations in facial video associated with cardiac blood-volume changes [1,2]. Thus, the input is a sequence of facial video frames, and the output in the present study is a scalar HR estimate in beats per minute for each 10 s segment. Because rPPG requires only a camera and no attached sensor, it enables contactless physiological monitoring when conventional contact sensors are inconvenient or undesirable. However, accurate rPPG estimation remains challenging because pulse-related color variations are weak and can be contaminated by motion, illumination changes, and camera-dependent artifacts.

Existing rPPG methods range from conventional signal-processing approaches to end-to-end deep-learning models. Conventional methods, including independent component analysis (ICA), the chrominance-based method (CHROM), and the plane-orthogonal-to-skin method (POS), recover pulse-related components from facial red–green–blue (RGB) traces using statistical or optical-model-based assumptions [3,4,5]. These methods are computationally compact and interpretable, and they remain important baselines for remote HR estimation.

Deep learning methods operate on raw facial videos, frame differences, spatiotemporal representations, or reconstructed physiological waveforms. Representative examples include DeepPhys, PhysNet, TS-CAN, EfficientPhys, and PhysFormer [6,7,8,9,10]. These models have substantially advanced rPPG estimation by learning spatial and temporal representations from video data. However, the internal representations and prediction mechanisms of high-capacity end-to-end models are often difficult to relate directly to explicit physiological quantities, such as cardiac frequency. Moreover, differences in input representations, learning objectives, model capacities, and evaluation protocols make it difficult to isolate which architectural components are responsible for the observed performance improvements.

In addition to these approaches, hybrid pipelines combine explicit signal extraction with learned feature processing. Such pipelines first derive a one-dimensional pulse-like signal using a conventional color-projection method and then transform it into a representation suitable for neural-network processing [11]. The present study adopts this approach by applying CHROM, followed by a continuous wavelet transform (CWT) and convolutional neural network (CNN)-based HR estimation. This controlled pipeline allows the effect of the CNN readout to be isolated while keeping the preceding signal extraction and time–frequency representation fixed.

A CWT scalogram is particularly relevant to HR estimation because it represents the temporal evolution of spectral energy while retaining an interpretable frequency axis. In this representation, the position of the cardiac component along the frequency axis directly corresponds to HR. The frequency coordinate is therefore not a nuisance variable but a physically meaningful component of the input representation. Moreover, the time–frequency structure allows transient non-cardiac components and changes in signal quality to be inspected over time. The CWT scalogram is not adopted under the assumption that it necessarily outperforms conventional fast Fourier transform (FFT)-based HR estimation. Rather, it provides a structured representation for investigating frequency-preserving readout design and tracing residual errors to the extracted input signal.

Global average pooling (GAP) averages each feature map across its spatial positions before classification or regression. This operation is useful in conventional image classification because the predicted class should generally not depend on where an object appears in an image. However, the axes of a CWT scalogram are not merely spatial coordinates. The vertical axis represents frequency, and the position of the cardiac component along this axis directly corresponds to HR. When GAP averages features across the frequency axis, this position is no longer passed explicitly to the regression head. The preceding convolutional layers may retain some frequency-position information indirectly within their feature channels, but the final HR estimate must then recover the frequency from this implicit representation. A readout that retains the frequency axis may therefore be better aligned with the structure of this task.

Previous studies have applied learning-based models to time–frequency representations of physiological signals, but the importance of retaining the frequency coordinate in the readout has received limited systematic investigation. In particular, it remains unclear whether a frequency-preserving readout improves HR estimation when the input representation, CNN backbone, training protocol, and evaluation splits are held fixed. It is also unclear whether the remaining errors originate from the learned readout or from artifacts already present in the extracted pulse-like signal.

Accordingly, the primary objective of this study is to improve the readout design for CWT-based rPPG heart-rate estimation by retaining the physically meaningful frequency-coordinate structure until the final prediction stage, rather than collapsing it prematurely through conventional scalar regression. A second objective is to improve the physiological traceability of the estimation process and to characterize the conditions under which the method succeeds or fails, including HR-range mismatch, motion, illumination reduction, and cross-dataset transfer.

**Figure 1:**
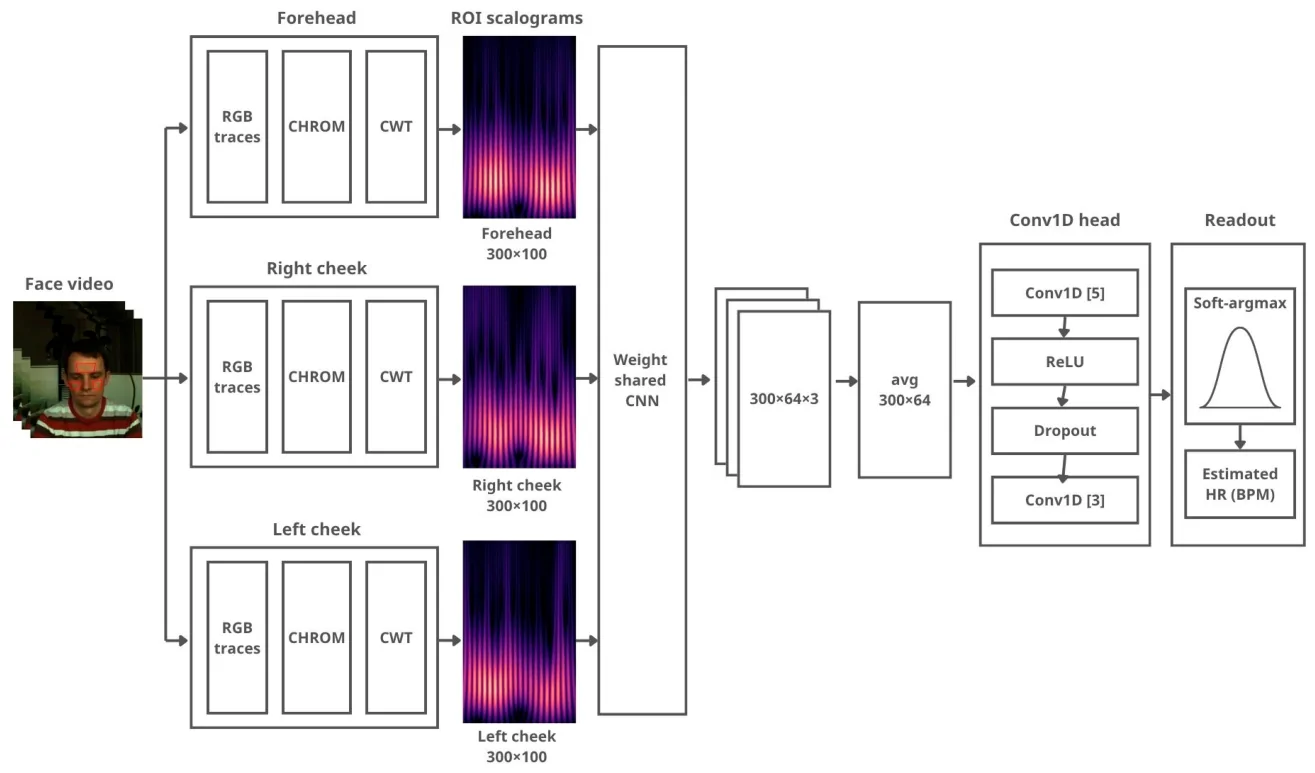
Overview of the proposed three-ROI rPPG heart-rate estimation framework. Facial video is divided into three regions of interest (forehead, right cheek, and left cheek), from which RGB traces are extracted and converted to CHROM signals. Continuous wavelet transform (CWT) is then applied to generate a 300 × 100 scalogram for each ROI. The three scalograms are processed by a weight-shared CNN, producing frequency-preserving feature representations of size 300 × 64 for each ROI. These representations are combined by element-wise averaging, followed by a Conv1D readout head. The final soft-argmax readout converts the frequency-bin scores into a continuous heart-rate estimate in beats per minute (BPM), while retaining the explicit correspondence between the frequency axis and the output HR coordinate. The numbers in square brackets in the Conv1D blocks indicate kernel sizes.

To address the first question, we develop a CNN that retains the frequency axis and produces a score for each predefined beats-per-minute (BPM) coordinate. Soft-argmax converts the resulting BPM score profile into a continuous HR estimate. This creates a physiologically traceable correspondence among the frequency structure of the input scalogram, the learned BPM score profile, and the final HR estimate. We compare this frequency-preserving readout with GAP and several alternative readouts while holding the input pipeline, CNN backbone, training procedure, and leave-one-subject-out (LOSO) splits fixed. A non-learned CWT-argmax baseline is also included to distinguish the contribution of CWT-based frequency localization from that of the learned soft-argmax readout. Figure 1 provides a visual overview of this processing flow and the relationship between the facial-video input, CWT representation, frequency-preserving CNN, and structured soft-argmax readout.

To address the second question, we examine whether large errors arise from limitations of the learned model, such as inadequate coverage of high HR values during training, or from artifacts already present in the extracted signals. We analyze the reference signals, individual color channels, CHROM-projected signals, and facial motion of the three UBFC-rPPG subjects with the largest errors. These analyses are treated as descriptive case studies of projection-sensitive, projection-persistent, and motion-associated failure patterns. We further evaluate error patterns under the controlled motion conditions provided by PURE and the controlled illumination conditions provided by BH-rPPG. Finally, we explore whether reference-free characteristics of the input scalogram can identify potentially unreliable estimates before an HR estimate is returned.

The main contributions of this study are as follows:

1. We demonstrate that, within the evaluated CHROM-CWT architecture, the complete structured soft-argmax readout provides a more reliable mapping from frequency-indexed features to HR than the matched GAP readout. Under matched experimental conditions, soft-argmax significantly reduced subject-level HR estimation error relative to GAP on both PURE and UBFC-rPPG. Because the structured readout couples explicit BPM-bin scoring, probabilistic normalization, coordinate-based expectation, and output-range restriction, this result is interpreted as evidence for the complete readout formulation rather than for frequency-coordinate preservation alone.
2. We progressively examine alternative explanations for the structured-readout advantage, including local Conv1D processing, output-range restriction, readout capacity, and direct non-learned frequency localization. The matched GAP controls use the same post-fusion Conv1D feature extractor as the proposed model, and removing this shared block did not change the relative ordering of the readouts on PURE. Together with the bounded-GAP and CWT-argmax controls, these analyses narrow the interpretation to the complete structured soft-argmax formulation rather than frequency-coordinate preservation alone.
3. We provide evidence that important residual errors originate from limitations or distortions already present in the extracted input signals, rather than solely from the CNN readout. Subject-level failure analyses identify errors associated with HR-range mismatch, color projection, non-cardiac periodic components, and facial motion. Controlled experiments further show that the evaluated CWT-based estimators exhibited smaller illumination-induced degradation than the evaluated FFT-based estimators, supporting the utility of the time–frequency representation for physiologically traceable failure analysis.

## 2. Related Work

We review four lines of research directly related to the contributions of this study: classical signal-processing-based rPPG estimation, video-based deep-learning models, CWT-based time–frequency learning, and soft-argmax-based differentiable coordinate estimation. This organization positions the proposed method relative to conventional color-projection pipelines, modern video-based models, previous CWT-based rPPG methods, and established coordinate-preserving readouts.

### 2.1. Classical Signal-Processing-Based rPPG

Classical rPPG methods extract a one-dimensional pulse signal from facial color traces using statistical or optical-model-based assumptions. Early approaches formulated pulse recovery as a blind source separation problem. Independent component analysis (ICA) [3], for example, separates statistically independent components from facial color traces and selects a component associated with cardiac activity.

Subsequent methods introduced projections based more directly on skin-reflection properties. The chrominance-based method (CHROM) proposed by de Haan and Jeanne [4] combines chrominance signals to suppress motion-related variations in specular reflection. The plane-orthogonal-to-skin method (POS) proposed by Wang et al. [5] projects temporally normalized RGB signals onto a plane orthogonal to the skin-tone direction, improving robustness to motion-induced intensity changes.

These methods remain important because they are computationally compact, physiologically interpretable, and reproducible. They are also used as front ends in hybrid pipelines, in which a pulse-like signal is first extracted from facial RGB traces and subsequently processed by a learning-based model. The present study follows this hybrid design by using CHROM as a fixed signal-extraction front end and examining the CNN readout applied after CWT-based time–frequency conversion.

### 2.2. Video-Based Deep Learning for rPPG

Video-based deep-learning methods learn physiological representations directly from facial videos or frame-derived inputs. DeepPhys [6] combines normalized frame differences with a two-branch convolutional attention network. PhysNet [7] uses three-dimensional convolutions to jointly model spatial and temporal information and reconstruct an rPPG waveform. TS-CAN [8] combines temporal shift modules with attention mechanisms to improve temporal modeling while maintaining computational efficiency. RhythmNet [12] converts facial video into a spatial–temporal map and combines CNN-based feature extraction with temporal modeling for end-to-end HR estimation.

More recent architectures have introduced transformer-based or efficiency-oriented designs. PhysFormer [10] uses temporal-difference transformers to model local and long-range spatiotemporal dependencies and incorporates frequency-domain supervision during training. EfficientPhys [9] reduces architectural complexity and computational cost to support efficient camera-based physiological measurement.

Self-supervised and unsupervised learning have also been explored to reduce dependence on paired facial videos and contact-based physiological reference signals. Gideon and Stent [13] use contrastive learning with frequency and temporal-smoothness priors to learn physiological representations from unlabeled videos. Contrast-Phys [14] generates multiple rPPG signals from different spatiotemporal regions and trains the model using spectral similarities within a video and differences between videos. Reproducible benchmarking frameworks have also been developed to compare video-based rPPG models under more consistent preprocessing and evaluation protocols [2,15].

Recent work has increasingly incorporated physiological frequency information into video-based models. In particular, FreqPhys [16] uses physiological band-pass filtering, learnable spectral modulation, adaptive spectrum selection, and frequency-preserving diffusion to guide rPPG waveform reconstruction. This work demonstrates that frequency-domain information can improve denoising and generalization in high-capacity waveform-reconstruction models.

Cross-dataset generalization has also become an important focus in rPPG research. Recent studies have addressed dataset-specific pulse-distribution bias, domain-generalized feature learning, domain adaptation to previously unseen noise, and motion-transfer augmentation [17,18,19,20].

Video-based deep-learning models have achieved strong reported accuracy by learning complex spatiotemporal representations directly from facial videos. However, the relationship among their internal features, cardiac frequency, and the final physiological estimate is not always explicit. Even when frequency-domain supervision or spectral priors are incorporated, as in PhysFormer and FreqPhys, the models are primarily designed to improve waveform reconstruction, robustness, or generalization rather than to provide a coordinate-level explanation of a direct HR estimate.

The present study therefore pursues a complementary objective: physiologically traceable HR estimation within a compact hybrid pipeline. The CHROM signal and its CWT scalogram provide inspectable intermediate representations, while the frequency-preserving readout produces a score profile over predefined BPM values before calculating the final HR. This structure allows the estimated HR to be related directly to the frequency content of the extracted input signal. It does not make every internal CNN feature fully interpretable, but it provides a more explicit physiological connection between the input representation, the readout, and the final estimate than conventional scalar regression.

Because the video-based models and the present method differ substantially in input representation, preprocessing, supervision target, model capacity, subject split, and evaluation protocol, their reported values are discussed as broader context rather than used for direct ranking. Controlled conclusions in this study are restricted to methods evaluated under the same CHROM-CWT pipeline and subject-independent LOSO protocol.

### 2.3. CWT-Based Time–Frequency Learning for rPPG

A separate line of research converts a one-dimensional or multichannel pulse-related signal into a two-dimensional time–frequency representation before neural-network processing. The continuous wavelet transform (CWT) has a long history in the analysis of nonstationary biomedical signals because it represents signal energy jointly over time and scale or frequency [21]. In rPPG, a CWT scalogram can reveal both the frequency position of a cardiac component and changes in that component over time.

The closest prior study is the time–frequency learning framework proposed by Das et al. [11]. Their method computes CWT scalograms from raw RGB traces using a Gauss-2 mother wavelet and processes them using an attention-based encoder–decoder CNN. The network reconstructs an rPPG waveform, from which HR is subsequently calculated through conventional peak detection. The method was evaluated on UBFC-rPPG, COHFACE, and ECG-Fitness.

The present study differs from Das et al. in both input construction and prediction target. We first apply CHROM to obtain a one-dimensional pulse-like signal and then compute its CWT scalogram. Rather than reconstructing an rPPG waveform, the proposed model directly estimates HR by localizing the cardiac response along a predefined BPM axis. This design allows the relationship between the frequency coordinate of the input representation and the final HR estimate to be examined explicitly.

An FFT represents the aggregate frequency content of the entire analysis window with a fixed global frequency resolution determined primarily by the window duration. For a 10 s window, the nominal Fourier-bin spacing is approximately 0.1 Hz (6 BPM), although interpolation or zero-padding can provide a denser sampling grid without increasing the underlying spectral resolution. In contrast, the CWT provides a scale-dependent time–frequency representation: its effective time and frequency resolutions vary with scale, allowing transient spectral disturbances to be localized in time while persistent cardiac-frequency structure can be followed across the window. This does not mean that the CWT intrinsically suppresses noise or always provides higher frequency resolution than the FFT. Rather, its relevant advantage in the present setting is temporal localization of frequency content. The CWT scalogram is therefore used here as a structured representation for frequency-aware readout and for inspecting how cardiac-frequency structure changes under motion or illumination degradation. The dense CWT coordinate grid used in this study should likewise not be interpreted as equivalent spectral resolution.

Existing CWT-based rPPG work has primarily focused on reconstructing a pulse waveform from scalogram inputs, followed by conventional HR calculation. The design of a direct HR readout from a CWT scalogram—and specifically whether that readout should preserve the physically meaningful frequency coordinate—has received limited direct investigation. The present study isolates this question through matched backbone comparisons between GAP-based regression and a frequency-preserving soft-argmax readout.

### 2.4. Soft-Argmax and Differentiable Coordinate Estimation

Soft-argmax is a differentiable coordinate-estimation operator widely used outside the rPPG literature. In pose and landmark estimation, it converts a spatial score map into a continuous coordinate by applying softmax to the score values and computing the expectation over the corresponding coordinates [22,23]. Unlike hard argmax, which selects only the coordinate with the highest score, soft-argmax allows gradients to propagate through the coordinate-estimation operation.

The differentiable spatial-to-numerical transform (DSNT) proposed by Nibali et al. [24] formalized a closely related coordinate-regression approach. DSNT converts normalized spatial activations into numerical coordinates without requiring a fully connected regression layer and introduces regularization to encourage well-formed spatial distributions.

These approaches are typically used to localize landmarks or key points in spatial score maps. The same principle is applicable to CWT-based HR estimation because the target can be represented as a coordinate along the frequency axis. In the proposed model, the CNN produces a one-dimensional score profile over predefined BPM values. Softmax converts these scores into a probability distribution, and soft-argmax calculates the expected BPM value.

The contribution of this study is not the soft-argmax operation itself. Rather, it is the controlled evaluation of a frequency-preserving, differentiable coordinate readout within a CWT-based rPPG pipeline. By comparing this readout with GAP-based regression, range-constrained GAP, alternative regression heads, and non-learned CWT-argmax under matched conditions, we examine whether preserving the physically meaningful frequency coordinate improves HR estimation.

## 3. Materials and Methods

This section first describes the datasets and signal preprocessing (Section 3.1, Section 3.2 and Section 3.3), followed by the proposed method in Section 3.4, entitled “Frequency-Preserving CNN with Soft-Argmax Readout.” Section 3.4 constitutes the proposed approach: a frequency-preserving shared CNN backbone, cross-ROI fusion, and a structured soft-argmax readout. The subsequent sections describe the training configuration (Section 3.5), baseline and control methods used for comparison (Section 3.6), and the evaluation protocol and statistical analysis (Section 3.7).

### 3.1. Datasets and Subject Inclusion Criteria

We used three public rPPG datasets: PURE [25], UBFC-rPPG [26], and BH-rPPG [27]. PURE and UBFC-rPPG were used for the primary within-dataset evaluation and architectural analyses. BH-rPPG was used as a complementary controlled analysis of illumination-related degradation.

PURE contains 10 subjects recorded under six controlled conditions, giving 60 sessions in total. The videos were recorded at 30 frames/s with a resolution of 640 × 480 pixels and a duration of approximately 60 s per session. The reference signal was acquired using a Pulox CMS50E pulse oximeter sampled at 60 Hz. The six recording conditions were Steady, Talking, Slow Translation, Fast Translation, Small Rotation, and Medium Rotation. These conditions were used in the motion-condition analysis described in Section 4.4.

UBFC-rPPG is distributed as 44 subject directories containing facial videos recorded at 30 frames/s and measurements from a contact pulse oximeter. In the release used in this study, subject21 and subject28 could not be analyzed because the required reference data were unavailable.

Among the remaining 42 subjects, subject11, subject18, subject20, and subject24 were excluded through a post hoc reference-quality screening procedure. For each subject, we calculated the standard deviation of the segment-level mean reference HR across the analyzed 10 s windows. Four subjects showed markedly larger HR variability than the rest of the cohort, with standard deviations of 31.6, 32.2, 36.4, and 44.1 BPM. In contrast, the standard deviations among the other 38 subjects ranged up to 12.0 BPM, with a mean of 3.4 ± 2.6 BPM.

Because no subjects had values between 12.0 and 31.6 BPM, a threshold of 15 BPM was used to separate the four markedly atypical reference traces from the remaining cohort. This threshold was selected after inspection of the subject-level reference-HR variability and was not prespecified before analysis. The exclusion was based solely on the reference pulse-oximeter signal and was performed independently of the camera-derived signals and model predictions.

These procedures yielded a 38-subject UBFC-rPPG cohort, which was used consistently in the primary UBFC-rPPG experiments unless otherwise stated. Because the 15 BPM threshold was selected post hoc, we additionally performed an exclusion-sensitivity analysis in which the principal soft-argmax-versus-matched-GAP comparison was repeated using all 42 subjects with usable reference signals.

BH-rPPG contains 35 subjects recorded under low, medium, and high illumination. It was used to examine how controlled changes in illumination affected the preprocessed signal, the CWT scalogram, and the resulting HR estimates. Because the effective frame rate varied across recordings, segmentation and synchronization were based on the recorded timestamps rather than on an assumed constant frame rate. No BH-rPPG subject was excluded from the analysis.

### 3.2. Preprocessing Pipeline

#### 3.2.1. Region-of-Interest Extraction

MediaPipe FaceMesh [28] was used to locate 468 facial landmarks in each frame. Three regions of interest (ROIs)—the forehead, right cheek, and left cheek—were defined as convex polygons using fixed landmark indices. The indices follow the zero-based MediaPipe convention, as follows:

- Forehead: 67, 297, 296, and 66;
- Right Cheek: 116, 120, 203, and 147;
- Left Cheek: 349, 340, 411, and 423.

The cheek polygons covered the mid-cheek skin regions lateral to the nasolabial folds, whereas the forehead polygon covered the central forehead while avoiding the eyebrows and hairline as far as possible. The spatial mean of each RGB channel was calculated within each polygon in every frame, producing one RGB time series for each ROI.

#### 3.2.2. CHROM Signal Extraction

A pulse-like signal was extracted from the RGB trace of each ROI using CHROM [4]. Within every 1.5 s sliding window, the RGB signals were normalized by their corresponding temporal means:

(1) $$r_{n}(t)=R(t)/\mu_{R}\;g_{n}(t)=G(t)/\mu_{G}\;b_{n}(t)=B(t)/\mu_{B}$$

where $\mu_{R}$, $\mu_{G}$ and $\mu_{B}$ denote the mean values of the corresponding color channels within the window. Two chrominance signals were then calculated as

(2) $$X_{s}(t)={3r}_{n}-{2g}_{n}\;Y_{s}(t)={1.5r}_{n}+g_{n}-{1.5b}_{n}$$

The CHROM pulse signal was obtained as

(3) $$S=X_{s}(t)-{\alpha Y}_{s}(t)\;\alpha ={\sigma (X}_{s})/{\sigma (Y}_{s})$$

where σ(⋅) denotes the standard deviation calculated within the corresponding temporal window.

#### 3.2.3. Post-Processing and Segmentation

Each extracted pulse signal was filtered using a fourth-order zero-phase Butterworth band-pass filter with a passband of 0.75–2.5 Hz, corresponding to 45–150 BPM. The filtered signal was then detrended and z-score normalized.

The signals were divided into 10 s segments with a stride of 0.5 s. To reduce boundary effects from filtering, the first and last 5 s of each recording were removed before segmentation. For PURE and UBFC-rPPG, the 5 s trimming corresponded to 150 frames at 30 frames/s.

#### 3.2.4. Reference HR Generation

The reference HR for each segment was derived from the HR values provided by the corresponding dataset or reference device. We did not independently estimate reference HR by applying peak detection or inter-beat interval analysis to the reference waveform.

For PURE, the pulse-rate trace provided by the pulse oximeter was aligned to the video-frame timestamps by linear interpolation. Values outside the available timestamp range were assigned the nearest endpoint value. For UBFC-rPPG, the dataset-provided per-frame HR trace was used directly without additional interpolation. For BH-rPPG, the provided reference HR was synchronized with the video using the recorded timestamps. Timestamp-based synchronization was also used to define the 10 s segments because the effective video frame rate was not constant.

After temporal alignment and boundary trimming, the reference HR trace was segmented using the same 10 s windows and 0.5 s stride as the input signal. The reference HR assigned to each segment was the arithmetic mean of the available HR values within that segment. No additional peak detection, inter-beat interval estimation, temporal smoothing, or segment-level outlier removal was applied. The subject-level reference-quality screening for UBFC-rPPG was performed separately, as described in Section 3.1.

### 3.3. CWT Scalogram Generation

For each 10 s segment and each ROI, a continuous wavelet transform (CWT) scalogram was computed from the extracted CHROM signal. For a one-dimensional signal x(t), the CWT is defined as

(4) $$W_{x}\left(a,b\right)=\int_{-\infty }^{\infty }x(t)\frac{1}{\sqrt{a}}\psi \;*\;\left(\frac{t-b}{a}\right)dt$$

where a denotes the scale, b denotes the temporal shift, ψ is the mother wavelet, and ∗ denotes complex conjugation. A real-valued Morlet wavelet was used, and the scalogram was defined as the magnitude of the CWT coefficients, $|W_{x}(a,b)|$.

The transform was implemented using PyWavelets 1.7.0 (pywt.cwt) with the real-valued Morlet wavelet ‘morl’, defined as ψ(t) = exp(−t^2^/2) cos(5t). In the PyWavelets implementation, this wavelet is evaluated over an effective support of t ∈ [−8, 8] and is real-valued (complex_cwt = False). Accordingly, the scalogram used here is the magnitude of real-valued CWT coefficients rather than the magnitude of an analytic complex transform.

Before CWT computation, each 10 s CHROM signal was resampled to 60 Hz, producing a fixed-length sequence of 600 samples. For PURE and UBFC-rPPG, the RGB-derived signals were resampled from 30 frames/s. For BH-rPPG, timestamp-based resampling was used because the effective video frame rate was not constant. This procedure standardized the signal length and temporal sampling grid used for CWT computation without introducing physiological information beyond that contained in the original video signal.

The CWT scales were selected to correspond to 300 target frequencies linearly spaced between 0.75 and 2.5 Hz, equivalent to 45–150 BPM. The resulting frequency spacing was

(5) $$∆f=\frac{2.5-0.75}{300-1}=0.00585\;Hz=0.35BPM\;per\;Bin$$

For each target frequency $f_{i}$, the corresponding scale was calculated using the scale-to-frequency relationship of the Morlet wavelet:

(6) $$a_{i}=\frac{f_{c}f_{s}}{f_{i}}$$

where $f_{s}=60$ Hz is the resampled sampling frequency and $f_{c}$ is the central frequency of the selected Morlet wavelet.

For scale conversion, the normalized central frequency was fc = 0.8125. With fs = 60 Hz, the 300 target frequencies from 0.75 to 2.50 Hz corresponded to scales from approximately 65.00 to 19.50, respectively.

The 300 target frequencies therefore sample the predefined frequency coordinate at intervals of approximately 0.00585 Hz (0.351 BPM). This is the same 300-point coordinate grid used in Equation (9): Δb = (150 − 45)/(300 − 1) = 0.351 BPM/bin, which is equivalent to 60Δf for the spacing Δf defined in Equation (5). This spacing is the sampling density of the CWT frequency axis and should not be interpreted as the effective spectral resolution of a 10 s segment. Effective resolution is governed by the Morlet wavelet’s time–frequency bandwidth together with the finite observation window; the latter alone provides a characteristic frequency scale of approximately 1/T = 0.1 Hz (6 BPM) for T = 10 s. Thus, the dense 300-bin axis provides a finely sampled coordinate system for the soft-argmax readout rather than an ability to resolve spectral components separated by 0.351 BPM.

Each initial scalogram had dimensions of 300×600, corresponding to frequency bins and time samples, respectively. To reduce computational cost while preserving the complete frequency axis, the scalogram was resized only along the temporal dimension, from 600 to 100 samples, using area interpolation. The resulting 300×100 scalogram was independently standardized for each ROI using z-score normalization over all scalogram elements. Each 10 s segment was therefore represented by three ROI-specific scalograms of size 300×100.

### 3.4. Frequency-Preserving CNN with Soft-Argmax Readout

**Figure A1:**
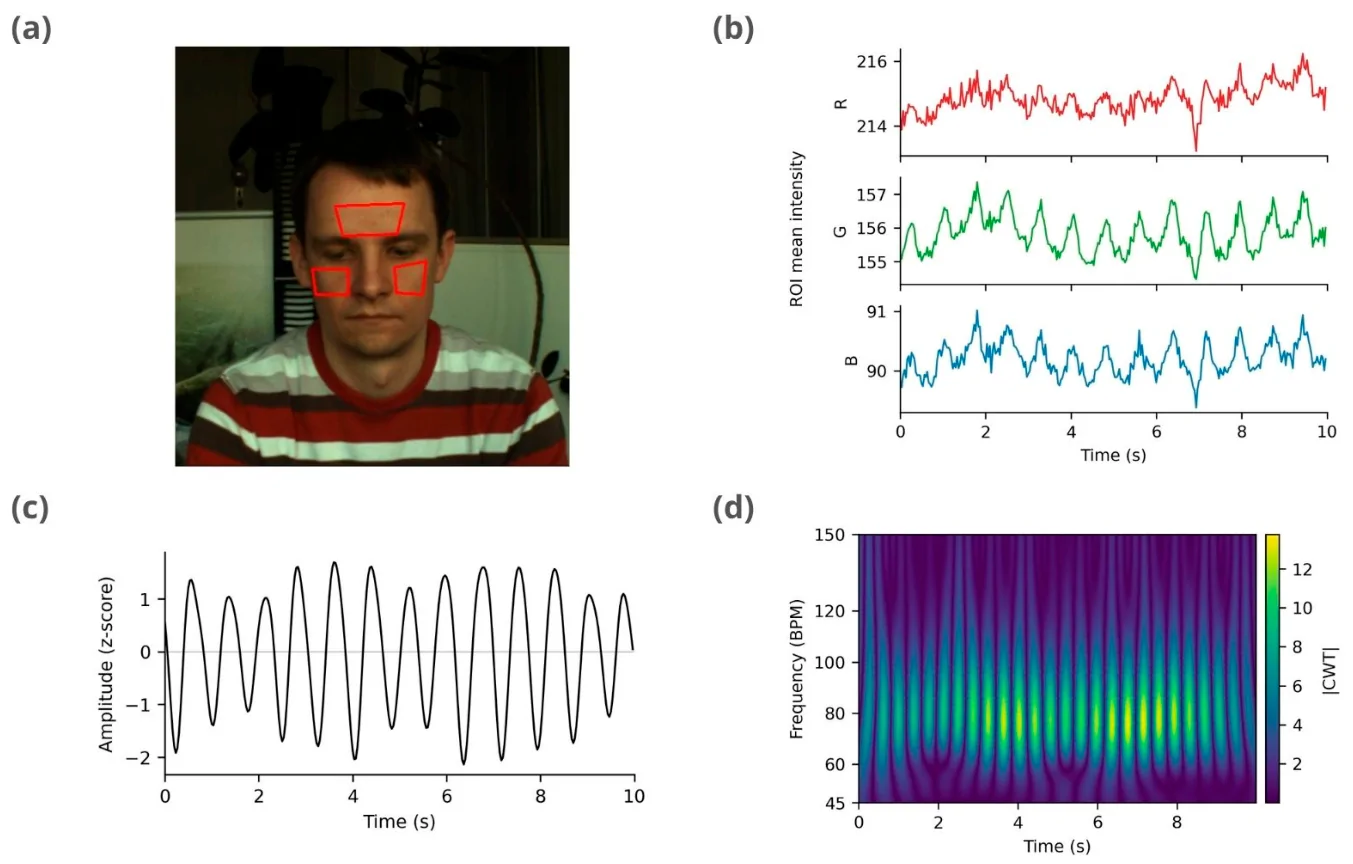
Representative example of the dataset input and intermediate signal representations used in the proposed pipeline. (**a**) Representative facial video frame with the forehead, right-cheek, and left-cheek regions of interest (ROIs). (**b**–**d**) Representative signals derived from the forehead ROI shown in panel (**a**). (**b**) Temporal red-green-blue (RGB) intensity traces over a 10 s segment. (**c**) Corresponding z-scored CHROM signal. (**d**) Continuous wavelet transform (CWT) scalogram derived from the CHROM signal, represented over time and BPM-frequency coordinates.

The proposed method is described in this subsection and summarized in Figure 1, which shows the complete rPPG heart-rate estimation pipeline and the central readout design evaluated in this study. Facial video is first divided into forehead, right-cheek, and left-cheek regions of interest (ROIs). Temporal red–green–blue (RGB) traces from each ROI are converted to pulse-like signals using CHROM and then to CWT scalograms. A representative example of the facial-video input, ROI definition, RGB traces, CHROM signal, and resulting CWT scalogram is provided in Appendix B (Figure A1). The three scalograms are processed by a weight-shared CNN that preserves the frequency dimension, fused across ROIs, and mapped to a continuous heart-rate estimate by the structured soft-argmax readout. The central design principle is to retain the explicit frequency/BPM coordinate until the final prediction stage rather than collapsing it before scalar regression.

#### 3.4.1. Shared ROI Backbone

Each ROI-specific 300×100 scalogram was processed by a CNN backbone with weights shared across the three ROIs. The first spatial dimension represented frequency, and the second represented time. The backbone consisted of six Conv2D blocks, each followed by batch normalization and a ReLU activation. The first four convolutional layers used 3×3 kernels, whereas the final two used 3×5 kernels to provide a wider receptive field along the temporal axis. The numbers of output channels were 16, 16, 32, 32, 64, and 64, respectively.

Downsampling was applied only along the temporal axis. Starting from an input temporal length of 100, the temporal length after each of the six convolutional blocks was 50, 50, 25, 25, 5, and 1, respectively.

**Figure 2:**
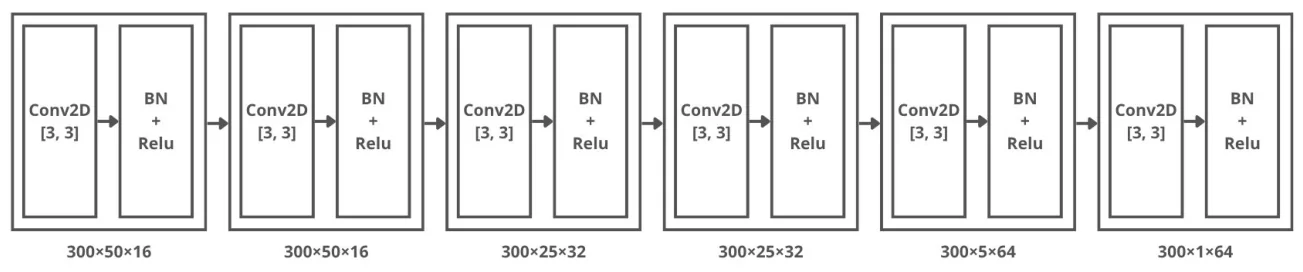
Detailed architecture of the shared frequency-preserving CNN backbone. The backbone comprises six Conv2D–batch normalization–ReLU blocks with output dimensions of 300 × 50 × 16, 300 × 50 × 16, 300 × 25 × 32, 300 × 25 × 32, 300 × 5 × 64, and 300 × 1 × 64, respectively. Downsampling is applied only along the temporal axis, thereby preserving the complete 300-bin frequency axis throughout the network.

The frequency dimension was not downsampled and remained at 300 bins throughout the backbone. This design retained an explicit frequency coordinate for the subsequent structured readout, while the convolutional kernels were allowed to integrate information locally across neighboring frequency bins. Such local frequency-domain receptive fields were intended to capture the smooth ridge structure of the CWT representation rather than to treat each sampled frequency coordinate as independent. Each ROI branch therefore produced a 300 × 64 feature representation. Because the same backbone parameters were used for all three ROI inputs, corresponding features were extracted under a common representation. Figure 2 summarizes the detailed architecture of the shared backbone, including the feature-map dimensions at each convolutional block and the temporal-only downsampling that preserves the full 300-bin frequency axis.

#### 3.4.2. Cross-ROI Fusion

The three ROI-specific feature representations were averaged element-wise to produce a single 300 × 64 fused representation. To evaluate whether more flexible cross-ROI feature interactions improved this fusion, we additionally implemented a cross-attention module as a controlled architectural comparison. In the cross-attention variant, the three ROI-specific 300 × 64 feature representations were processed using three pairwise multi-head attention blocks with four heads and key and value dimensions of 16. The 300 frequency bins, rather than the three ROIs themselves, were treated as attention tokens; thus, the frequency-bin representation of one ROI attended over the 300 frequency-bin representations of another ROI, yielding a 300 × 300 attention map for each head. Each attention output was layer-normalized, added to the corresponding query representation through a residual connection, and normalized again. The three resulting pairwise 300 × 64 representations were summed element-wise to obtain the final 300 × 64 fused representation, which was passed to the unchanged soft-argmax head. Only the fusion operation was changed; the CHROM-CWT inputs, shared frequency-preserving backbone, soft-argmax readout, preprocessing, training protocol, and LOSO splits were held fixed. The cross-attention fusion module added 50,688 trainable parameters, increasing the complete model size from 119,921 to 170,609 parameters. Attention selectivity was characterized using normalized attention entropy, for which a value of 1.0 denotes a uniform distribution, and by examining correlations between attention statistics and input-quality measures. We also compared subject-level MAE and performance across the controlled PURE motion conditions to determine whether cross-attention was beneficial in specific subjects or recording conditions. The quantitative comparison is reported in Section 4.2.5.

#### 3.4.3. Soft-Argmax Readout

The fused 300×64 representation was passed to a one-dimensional prediction head operating along the frequency axis. The head consisted of a Conv1D layer with 32 filters and a kernel size of 5, followed by a ReLU activation, a dropout layer with a rate of 0.3, and a final Conv1D layer with one filter and a kernel size of 3. The frequency dimension was preserved, producing one score $z_{i}\;$ for each of the 300 frequency bins.

The scores were converted into a probability distribution over the frequency bins using softmax:

(7) $$p_{i}=\frac{exp(\frac{z_{i}}{\tau })}{\sum_{j=1}^{300}exp(\frac{z_{j}}{\tau })}$$

where τ denotes the softmax temperature. The final HR estimate was calculated as the expected BPM coordinate under this distribution:

(8) $$\hat{HR}=\sum_{i=1}^{300}p_{i}b_{i}$$

where the BPM coordinate of the i-th frequency bin was defined as

(9) $$b_{i}=60f_{i}=45+\left(i-1\right)\frac{150-45}{300-1},\;i=1\ldots 300$$

The softmax temperature was fixed at τ=1. Because the estimate was calculated as a weighted average of the BPM coordinates, the output was continuous within the predefined 45–150 BPM range rather than restricted to one of the discrete frequency bins. The CNN backbone and soft-argmax readout were trained jointly by backpropagation.

Conceptually, this readout differs from global-average-pooling regression in the point at which the frequency coordinate is removed. In the proposed formulation, the network first produces one score for each BPM-indexed frequency bin, normalizes the score profile, and computes the expected BPM coordinate. Global average pooling, by contrast, collapses the frequency-indexed representation before the final scalar regression. Figure 1 illustrates this frequency-preserving path from the CWT scalogram to the final HR estimate.

### 3.5. Training Configuration

All learned models were trained using the Huber loss with δ = 5.0 and the Adam optimizer with an initial learning rate of $5\times 10^{-4}$. The learning rate was reduced by a factor of 0.5 when the validation loss did not improve for seven consecutive epochs. The batch size was 16, and training continued for a maximum of 80 epochs. Early stopping was applied with a patience of 15 epochs, and the weights from the epoch with the lowest validation loss were restored.

Within each LOSO fold, one subject was held out for testing and the validation subject was chosen deterministically from the remaining non-test subjects. Specifically, the mean reference HR was calculated for each non-test subject, the median of these subject-level mean HR values was obtained, and the subject whose mean reference HR was closest to this median was assigned to validation. This rule involved no random selection. Thus, for PURE, each fold contained eight training subjects, one validation subject, and one test subject.

The primary experiments were implemented in Python 3.10.11 using TensorFlow 2.10.1, Keras 2.10.0, NumPy 1.26.4, SciPy 1.15.2, PyWavelets 1.7.0, MediaPipe 0.10.32, OpenCV 4.11.0, scikit-learn 1.5.1, and pandas 2.0.1. A seed value of 42 was used for the fold ordering and for the shuffling and augmentation of training segments (with fold-specific offsets where applicable). However, the TensorFlow global random seed was not set in the primary training scripts; therefore, weight initialization and dropout masks were not fully fixed. Unless otherwise stated, the primary results correspond to one complete LOSO run with one training initialization per fold rather than to averages over multiple initializations. The additional random-seed sensitivity analysis described in Section 3.7.3 was performed separately with seeds 42, 43, and 44, with the TensorFlow seed explicitly controlled, and is reported as a sensitivity analysis rather than being combined with the primary results.

For UBFC-rPPG, the training pipeline applied a reference-HR inclusion range of 50–130 BPM. In the retained 38-subject cohort, however, the empirically observed reference-HR range was 58.3–124.6 BPM; therefore, this restriction did not remove any segments from the retained cohort.

### 3.6. Baseline Methods

We evaluated two classical FFT-based estimators, a conventional GAP-based CWT-CNN, a matched backbone GAP variant, a bounded-output GAP control, a global max pooling (GMP) readout, a dense regression readout, and a non-learned CWT-argmax estimator. These baselines were selected to compare the proposed method with classical spectral peak selection, average- and max-pooling-based scalar regression, unconstrained dense regression, and non-learned frequency localization using the same CWT representation.

#### 3.6.1. CHROM-FFT

For each ROI, the FFT was applied to the same filtered CHROM segment used to construct the CWT input. The dominant spectral peak within 0.75–2.5 Hz was converted to BPM. The final estimate was the arithmetic mean of the estimates obtained from the three ROIs. Because this baseline uses the same CHROM projection, filtered segment, ROI definitions, and evaluation windows as the proposed CHROM-CWT pipeline, CHROM-FFT provides the matched FFT comparison for examining the effect of changing the spectral representation from CWT to FFT.

#### 3.6.2. POS-FFT

The plane-orthogonal-to-skin (POS) method of Wang et al. [5] was applied to the RGB trace of each ROI using a 1.6 s sliding window. The resulting POS signal underwent the same band-pass filtering, detrending, normalization, segmentation, and three-ROI averaging procedure used for CHROM-FFT. HR was estimated from the dominant FFT peak between 0.75 and 2.5 Hz. POS-FFT was included as an additional classical reference to examine whether observed error patterns were shared by another established color-projection method. Because POS-FFT changes the color-projection method and its projection-window configuration (1.6 s for POS versus 1.5 s for CHROM), it is not interpreted as a matched single-factor ablation.

#### 3.6.3. Conventional GAP-Based CWT-CNN

The conventional CWT-CNN received the same three CHROM-CWT scalograms as the proposed method. Its convolutional feature extractor was followed by global average pooling, a dense layer with 64 units, and a scalar HR-regression output. Global average pooling collapsed the spatial coordinates before scalar regression, so the resulting representation did not retain an explicit BPM-position axis. This model served as a broader GAP-based CNN baseline; because its backbone differed from the proposed model, the matched variant described below provided the direct readout comparison.

#### 3.6.4. Matched Backbone GAP Readout

To isolate the effect of readout design from that of the convolutional backbone, we evaluated a matched GAP variant. This model used the same CHROM-CWT inputs, shared frequency-preserving backbone, temporal downsampling schedule, cross-ROI averaging, and post-fusion Conv1D feature extractor as the proposed model. Specifically, the fused 300 × 64 representation was processed by a Conv1D layer with 32 filters and a kernel size of 5, followed by ReLU activation and dropout (0.3), producing a 300 × 32 feature map. This feature map was then averaged over the frequency axis using GlobalAveragePooling1D, passed through a 64-unit dense layer with ReLU activation and dropout (0.3), and mapped to a scalar HR estimate by a single linear output unit. All preprocessing, LOSO splits, validation-subject selection, optimization settings, random-seed configuration, and computational conditions were identical to those of the soft-argmax model. Thus, the matched GAP and proposed models shared the same local post-fusion Conv1D feature extractor and differed primarily in how the resulting frequency-indexed features were reduced to the final HR estimate.

#### 3.6.5. Bounded-Output GAP Control

To test whether the performance difference between GAP and soft-argmax could be explained solely by their output ranges, we evaluated a bounded GAP control on PURE and UBFC-rPPG. This model used the same CHROM-CWT inputs, frequency-preserving backbone, cross-ROI averaging, and post-fusion Conv1D feature extractor as the matched GAP model. The fused 300 × 64 representation was processed by Conv1D (32, kernel size 5), ReLU, and dropout (0.3), followed by GlobalAveragePooling1D, a 64-unit dense layer with ReLU activation and dropout (0.3), and a single-unit dense output. The resulting scalar z was transformed as 45 + 105σ(z), where σ denotes the sigmoid function. Predictions were therefore restricted to 45–150 BPM, matching the range of soft-argmax, while the readout did not explicitly retain the frequency coordinate. All preprocessing, LOSO splits, validation-subject selection, optimization settings, random-seed configuration, and computational conditions were identical to those used for the other matched-backbone models within each dataset. This control matched the shared local Conv1D processing and output range, but it was not constructed as a fully parameter-matched scalar head for a component-by-component decomposition of the soft-argmax formulation.

#### 3.6.6. Dense Regression Readout

The dense regression variant used the same frequency-preserving backbone and cross-ROI averaging as the proposed model. The fused 300 × 64 representation was processed by a Conv1D layer with 32 filters and a kernel size of 5, followed by a ReLU activation, producing a 300 × 32 feature map. This feature map was flattened into a 9600-dimensional vector and passed through a fully connected layer with 64 units and a ReLU activation. Dropout was then applied at a rate of 0.3, followed by a single linear output unit that produced an unconstrained HR estimate. Although flattening preserved the ordering of the frequency-indexed features, the readout did not explicitly map its prediction to the predefined BPM coordinates.

#### 3.6.7. Global Max Pooling Readout

The global max pooling (GMP) variant used the same CHROM-CWT inputs, frequency-preserving backbone, cross-ROI averaging, and post-fusion Conv1D feature extractor as the matched GAP model. The resulting frequency-indexed feature map was reduced using GlobalMaxPooling1D instead of GlobalAveragePooling1D, followed by the same scalar-regression layers. GMP therefore retained only the largest activation in each feature channel while discarding its frequency position and the remaining activation distribution.

#### 3.6.8. CWT-Argmax

The CWT-argmax baseline used the same CHROM-CWT scalograms as the proposed method but contained no learned parameters. Each ROI scalogram was averaged over time to obtain a frequency profile, and the three ROI profiles were averaged. The BPM coordinate of the largest response was used as the HR estimate. This baseline was included in the primary PURE and UBFC-rPPG comparisons and in the BH-rPPG illumination analysis to distinguish the effect of the CWT representation and explicit frequency localization from any additional effect of the learned soft-argmax readout.

#### 3.6.9. Frequency-Axis Resolution Sensitivity

To test whether retaining the full 300-bin frequency axis was necessary, we performed an additional controlled sensitivity analysis with the proposed soft-argmax architecture. The input scalogram was kept at 300 frequency bins, but frequency-axis strides were introduced within the backbone so that the fused representation and soft-argmax score profile contained 100 or 50 frequency coordinates instead of 300. The corresponding BPM-coordinate grids remained linearly spaced over 45–150 BPM. The convolutional filter counts, temporal downsampling schedule, ROI fusion, training settings, LOSO splits, and trainable parameter count (119,921) were kept unchanged across the 300-, 100-, and 50-bin variants. All three variants were rerun under the same computational environment so that the resolution comparison was internally matched. Subject-level differences relative to the 300-bin variant were evaluated using two-sided Wilcoxon signed-rank tests.

#### 3.6.10. HR-Distribution Coverage and Exploratory High-HR Augmentation Control

To characterize HR-range coverage and imbalance, we summarized the reference-HR distribution at the dataset and subject levels for PURE and UBFC-rPPG and evaluated performance in predefined HR bands. The bands were <60, 60–80, 80–100, 100–120, 120–140, and ≥140 BPM, with the final band omitted for UBFC-rPPG because no retained segment exceeded 140 BPM. For PURE, the corresponding segment counts were 2,363, 2,615, 236, 94, 554, and 28; for UBFC-rPPG, they were 44, 494, 1,282, 1,458, and 108 for the first five bands. For each HR band and method, we calculated the number of held-out test segments, MAE, RMSE, and the proportion of estimates within ±5 BPM using the existing LOSO test predictions; the complete stratified results are reported in Appendix A. Because the sub07 training fold contained no segments above 110 BPM, conventional oversampling of genuine high-HR segments was not possible. As an exploratory alternative, synthetic training examples were generated by rescaling the frequency axis of existing scalograms to shift their cardiac-frequency content. For each run, 50% additional training examples were generated with target HR values sampled over 45–150 BPM, while the original training data were retained. The sub07 fold was repeated for the proposed soft-argmax and matched GAP readouts using three random seeds (42, 43, and 44), with all other training settings unchanged. This augmentation experiment was treated as exploratory because frequency-axis rescaling cannot reproduce the full physiological and image-formation characteristics of genuine high-HR recordings.

### 3.7. Evaluation Protocol and Statistical Analysis

#### 3.7.1. Subject-Independent Evaluation

The learned models were evaluated using leave-one-subject-out (LOSO) cross-validation. In each fold, one subject was held out for testing, one of the remaining subjects was selected deterministically for validation according to the mean-HR median rule described in Section 3.5, and all other subjects were used for training. Each subject therefore appeared in the test set exactly once. The same train/validation/test assignments were used for models compared within each dataset.

PURE and UBFC-rPPG were used for the primary within-dataset evaluation. BH-rPPG was also evaluated using LOSO across all 35 subjects, but it was treated as a complementary controlled illumination analysis rather than as a general benchmark.

Cross-dataset generalization was evaluated without fine-tuning. A model trained using one primary dataset was evaluated on the other dataset without additional parameter updates. For the UBFC-rPPG-to-PURE direction, the PURE test segments were additionally stratified post hoc according to whether their reference HR fell below, within, or above the empirically observed UBFC-rPPG training range (58.3–124.6 BPM). These restricted-subset results were treated only as diagnostic decompositions; the results including all test subjects and segments were retained as the primary cross-dataset outcomes.

#### 3.7.2. Performance Measures

Performance was summarized using mean absolute error (MAE), root mean square error (RMSE), Pearson’s correlation coefficient (r), and the proportion of segments with an absolute error of no more than 5 BPM. Unless otherwise stated, descriptive performance values were pooled over all held-out test segments from the LOSO folds. Statistical comparisons between methods were conducted at the subject level so that overlapping segments from the same subject were not treated as independent observations.

To assess the effect of unequal subject weighting, we additionally calculated subject-macro-averaged MAE by first computing MAE separately for each held-out subject and then averaging the subject-level values so that each subject contributed equally. Subject-level MAE distributions were also summarized to characterize inter-subject variability.

To assess sensitivity to the 95% overlap between adjacent 10 s windows, we constructed non-overlapping test sets by retaining every twentieth segment within each recording. Because the original stride was 0.5 s, these yielded segments spaced 10 s apart without temporal overlap. All 20 possible starting offsets were evaluated. Model predictions were not recomputed because each segment was processed independently; the existing held-out predictions were subsampled according to each offset. Metrics and method rankings were then compared with those obtained from the full overlapping evaluation.

Agreement between estimated and reference HR was examined using Bland–Altman plots [29]. Because the analysis included overlapping segments and repeated observations from each subject, the limits of agreement were interpreted descriptively rather than as formal population-level inferential limits.

#### 3.7.3. Statistical Testing and Effect-Size Estimation

Differences between paired methods were evaluated using two-sided Wilcoxon signed-rank tests applied to subject-level MAE. Each pair consisted of MAE values from the same subjects under the same evaluation split. Segment-level observations were not used as independent samples in the significance tests. For the matched backbone readout comparison, GAP and soft-argmax were compared separately on PURE and UBFC-rPPG. The UBFC-rPPG bounded-output control was additionally compared with soft-argmax at the subject level. To distinguish the contribution of the learned structured readout from direct non-learned CWT frequency localization, soft-argmax was also compared with CWT-argmax at the subject level on both PURE and UBFC-rPPG. For paired comparisons, we reported the number of subjects favoring each method, the median paired MAE difference, a 95% bootstrap confidence interval obtained from 20,000 resamples of paired subjects with replacement, the Wilcoxon test statistic and *p* value, and rank-biserial correlation as an effect-size measure. Random-seed sensitivity for the UBFC-rPPG soft-argmax versus bounded-GAP comparison was assessed by repeating the complete 38-fold LOSO procedure with seeds 42, 43, and 44 while holding the code path, GPU environment, LOSO splits, optimization settings, and early-stopping criteria fixed; NumPy and TensorFlow seeds were explicitly controlled.

As a sensitivity analysis for the post hoc UBFC-rPPG reference-quality exclusion, we repeated the LOSO comparison between soft-argmax and the matched GAP readout using all 42 subjects with usable reference signals. The same comparison was also evaluated for the retained 38-subject cohort within this sensitivity-analysis run. We report segment-pooled MAE, the number of subjects favoring soft-argmax, the median paired subject-level MAE reduction, and the corresponding Wilcoxon signed-rank *p* value. These sensitivity results are reported separately from the primary 38-subject results and are used only to assess whether the exclusion changes the relative readout conclusion.

#### 3.7.4. Reference-Dependent and Reference-Free Input-Quality Analyses

Signal-quality indices have been used in contact and smartphone PPG to identify segments that support reliable physiological estimation [30,31]. More recently, this concept has been extended directly to remote PPG signals [32]. The exploratory analysis below examines whether temporal frequency instability and spectral entropy derived from the CWT scalogram are associated with absolute HR-estimation error.

To determine whether large estimation errors were associated with abnormalities already present in the preprocessed input signals, we conducted both reference-dependent diagnostic analyses and an exploratory reference-free quality analysis on UBFC-rPPG. For each 10 s segment, the CHROM spectra from the three ROIs were averaged, and the dominant frequency within 0.75–2.5 Hz was identified. For each subject, we calculated the proportion of segments in which the dominant CHROM frequency differed from the reference HR by more than 10 BPM. This measure was defined as the non-cardiac dominance score. Because the score did not use model predictions but required the reference HR, it was used only to assess whether high model errors were associated with dominant non-cardiac frequency components already present in the CHROM signal. Its association with subject-level MAE was summarized using Spearman’s and Pearson’s correlations. Agreement among estimators in identifying high-error subjects was evaluated using pairwise Cohen’s κ at the MAE thresholds reported in Section 4.3.

For the three UBFC-rPPG subjects with the largest MAEs for the proposed model, we additionally performed a descriptive signal-level analysis. The contact PPG provided with the dataset was transformed into the frequency domain to confirm that its dominant frequency was consistent with the supplied reference HR. Dominant in-band frequencies were then compared across CHROM, POS, the green channel, and components obtained by applying FastICA to the RGB traces. Rigid facial motion was assessed using MediaPipe ROI-centroid trajectories by calculating the temporal standard deviation of centroid displacement and examining spectral power near the dominant erroneous frequencies. Because each observed failure pattern was represented by only one subject, these analyses were interpreted as descriptive case studies rather than evidence of population-level failure mechanisms.

We also explored whether unreliable segments could be identified without access to the reference HR. For each UBFC-rPPG segment, temporal frequency instability was calculated as the standard deviation of the dominant BPM coordinate across time, and spectral entropy was calculated from the time-averaged frequency profile to quantify the dispersion of scalogram energy across frequencies. The two features were combined to define a quality score Q, with higher values indicating lower input quality. To prevent information from the held-out subject from influencing score calibration, standardization was performed separately within each LOSO fold: the means and standard deviations of the two quality features were estimated exclusively from the non-test training subjects and then applied unchanged to the held-out subject. For selective prediction, the rejection threshold corresponding to the highest 15% of Q values was likewise determined from the training-subject score distribution within each fold and then applied unchanged to the held-out subject. Thus, neither feature standardization nor threshold calibration used information from the held-out subject. The association between Q and absolute HR error was summarized using segment-level Spearman’s correlation. Because the 10 s windows overlapped, this correlation was interpreted descriptively rather than as an independent-sample statistical test. The feature combination and the 15% rejection proportion were selected post hoc on UBFC-rPPG and were not validated on an independent external dataset. Accordingly, this analysis was treated as exploratory and not as a validated deployment-ready rejection rule.

#### 3.7.5. BH-rPPG Illumination Analysis

BH-rPPG was evaluated using 35-subject LOSO cross-validation. Each subject contributed recordings under low, medium, and high illumination and appeared in the test set in exactly one fold. The same CHROM preprocessing, CWT frequencies, 10 s window length, and 0.5 s stride used for PURE and UBFC-rPPG were retained. Segments were defined using the recorded timestamps to account for variation in the effective frame rate.

Illumination-induced degradation was defined separately for each subject and method as the subject’s MAE under low illumination minus the corresponding MAE under high illumination. The degradation of the proposed model was compared with that of CWT-argmax, CHROM-FFT, and POS-FFT using paired two-sided Wilcoxon signed-rank tests and paired permutation tests. Holm’s correction was applied separately to the three planned comparisons for each test family.

Condition-specific method comparisons were performed at the subject level to help interpret the interaction results. The principal BH-rPPG claim was based on the paired high-to-low degradation contrasts rather than on segment-pooled differences alone.

To determine whether illumination effects were visible before the readout stage, the three ROI scalograms of each segment were averaged and summarized as a frequency profile. Changes in cardiac-band relative energy, peak prominence, spectral entropy, signal-to-noise ratio, and peak deviation from the reference HR were examined across illumination levels. The retrospective non-cardiac dominance score was also calculated at each illumination level. Correlations computed across only the three illumination levels were treated as descriptive and not as inferential statistical evidence.

## 4. Results

### 4.1. Primary Within-Dataset Results

Table 1 summarizes the primary within-dataset results on PURE and UBFC-rPPG. It compares the proposed frequency-preserving soft-argmax model with classical FFT-based estimators and the non-learned CWT-argmax baseline. Overall performance was calculated across all test segments, whereas statistical testing was performed at the subject level only for the prespecified method comparisons described in Section 3.7.3.

On UBFC-rPPG, the proposed model achieved lower MAE than CHROM-FFT (5.68 BPM), POS-FFT (4.79 BPM), and CWT-argmax (5.58 BPM). Relative to CWT-argmax, it reduced MAE from 5.58 to 3.82 BPM and RMSE from 11.80 to 6.70 BPM, while increasing the correlation coefficient from 0.78 to 0.91 and the within ±5 BPM rate from 77.5% to 79.7%. These results suggest that the learned readout provided a clearer benefit on UBFC-rPPG than on PURE.

On PURE, the proposed model achieved the lowest segment-pooled MAE (2.98 BPM), but its performance was numerically close to POS-FFT (3.02 BPM), CHROM-FFT (3.10 BPM), and CWT-argmax (3.10 BPM). Relative to CWT-argmax, the proposed model reduced RMSE from 8.89 to 6.00 BPM, indicating a reduction in the magnitude of large errors, whereas CWT-argmax achieved a higher within ±5 BPM rate (89.2% versus 87.1%). Thus, the proposed model did not uniformly outperform CWT-argmax across all PURE metrics, and its overall performance on PURE should be interpreted as comparable to the classical frequency-domain estimators rather than as a uniform improvement over them.

The non-learned CWT-argmax baseline achieved performance comparable to the FFT-based estimators on PURE, indicating that the CHROM-CWT representation itself retained sufficient frequency information for accurate HR estimation in many segments. The proposed readout showed a small segment-pooled MAE advantage on PURE and a clearer pooled improvement on UBFC-rPPG, motivating a paired subject-level comparison to determine whether the learned readout provided an additional benefit beyond direct CWT frequency localization.

At the subject level, the additional benefit of the learned readout differed between datasets. On UBFC-rPPG, soft-argmax achieved lower MAE than CWT-argmax in 25 of 38 subjects, with a median paired MAE reduction of 0.29 BPM (95% bootstrap CI, 0.02–0.67 BPM; Wilcoxon W = 197, *p* = 0.011; rank-biserial correlation = 0.47). RMSE showed a similar significant advantage (*p* = 0.022). On PURE, however, no significant subject-level difference was detected: CWT-argmax and soft-argmax had median subject-level MAEs of 2.25 and 2.32 BPM, respectively, and the median paired difference (CWT-argmax minus soft-argmax) was −0.03 BPM (95% CI, −0.31 to 0.53 BPM; W = 26, *p* = 0.922; rank-biserial correlation = 0.06). Thus, the small segment-pooled advantage of soft-argmax on PURE (2.98 versus 3.10 BPM) was not accompanied by a significant subject-level improvement, whereas an additional learned-readout benefit was evident on UBFC-rPPG.

We also examined the sensitivity of the UBFC-rPPG readout comparison to the four post hoc reference-quality exclusions. In a dedicated sensitivity run, the segment-pooled MAE for soft-argmax was 4.13 BPM for the retained 38 subjects and 4.94 BPM when all 42 usable subjects were included; the corresponding matched-GAP MAEs were 4.95 and 5.69 BPM. Including the four subjects therefore increased the absolute MAE of both readouts but did not change their relative ordering. At the subject level, soft-argmax remained significantly better than GAP in the retained cohort (26/38 subjects; median paired reduction, 0.91 BPM; *p* = 0.011) and in the full 42-subject cohort (28/42 subjects; median paired reduction, 0.86 BPM; *p* = 0.036). These results indicate that the post hoc exclusion affects the absolute performance values but does not alter the main relative conclusion of the matched readout comparison.

Because the methods in Table 1 differ in both feature extraction and readout design, this comparison does not isolate the effect of the readout head. A matched backbone comparison is therefore presented in Section 4.2.

**Figure 3:**
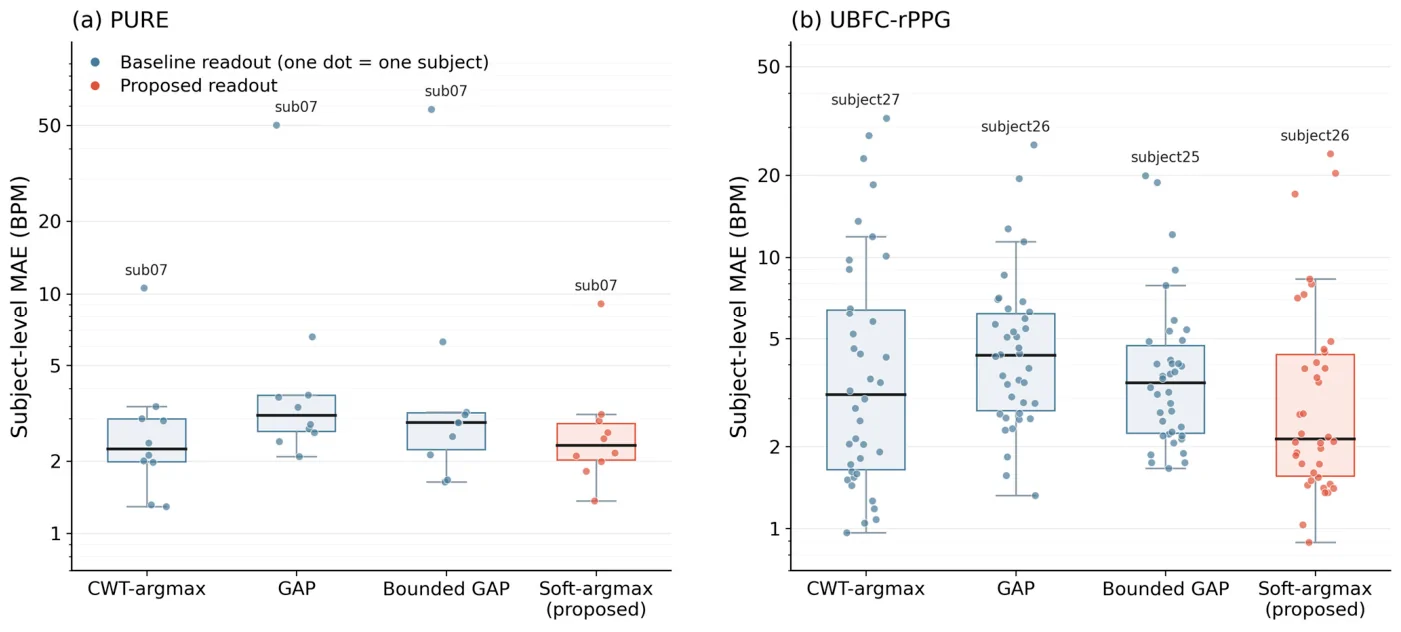
Distribution of subject-level MAE for the main readouts under leave-one-subject-out evaluation. (**a**) PURE (10 subjects) and (**b**) UBFC-rPPG (38 subjects). Each dot represents one subject. Boxes indicate the median and interquartile range, and whiskers extend to 1.5 × IQR. MAE was computed separately for each subject rather than pooled over segments. The subject with the largest error is labeled for each readout. The vertical axis is logarithmic because the worst-case subject reaches 50–58 BPM on PURE, whereas the medians lie near 2–4 BPM. On PURE, the four readouts have similar subject-level medians and differ mainly through the single out-of-range subject (sub07). On UBFC-rPPG, the proposed readout shifts the overall distribution downward (median 2.13 BPM versus 3.10–4.33 BPM for the baselines), while the worst-subject error remains of similar order.

Subject-macro averaging produced the same principal MAE ordering as the segment-pooled evaluation. On PURE, the macro-MAE was 2.97 BPM for the proposed method, 8.02 BPM for matched GAP, and 8.46 BPM for bounded GAP. On UBFC-rPPG, the corresponding values were 4.34, 5.55, and 4.57 BPM. Across the evaluated methods, the pooled-to-macro-MAE ranking was nearly identical on PURE (Spearman ρ = 0.991) and identical on UBFC-rPPG (ρ = 1.000). The subject-level distributions further showed that the pooled differences were influenced by a small number of high-error subjects, while the typical subject-level performance remained substantially lower than these extremes. The corresponding subject-level MAE distributions for the principal readouts are shown in Figure 3.

The non-overlapping sensitivity analysis yielded the same conclusion. Across all 20 possible non-overlapping tilings, the mean MAE differed from the original overlapping estimate by no more than 0.02 BPM for any evaluated method on either dataset. Rankings were unchanged for MAE, RMSE, Pearson’s r, within ±5 BPM, and subject-macro-MAE (Spearman ρ = 1.000 in all cases). The proposed readout also achieved lower MAE than the matched GAP readout in all 20 tilings on both PURE and UBFC-rPPG.

### 4.2. Frequency-Preserving Readout Design

#### 4.2.1. Matched Backbone Readout Comparison

We next evaluated the effect of readout design while holding the convolutional backbone fixed. The CHROM-CWT input, frequency-preserving backbone, ROI fusion, shared post-fusion Conv1D feature extractor, training protocol, and LOSO splits were identical across the matched GAP, bounded-GAP, and soft-argmax variants; the principal difference was the final reduction mechanism. Table 2 summarizes the matched backbone comparison of GAP, global max pooling (GMP), dense regression, and soft-argmax.

Replacing soft-argmax with GAP increased the segment-pooled MAE from 2.98 to 8.09 BPM on PURE and from 3.82 to 5.14 BPM on UBFC-rPPG. The advantage of soft-argmax was also observed for most subjects. On UBFC-rPPG, soft-argmax achieved a lower MAE than GAP in 31 of 38 subjects. The median subject-level MAE was 4.33 BPM for GAP and 2.13 BPM for soft-argmax, with a median paired reduction of 0.85 BPM (95% bootstrap CI, 0.58–1.78 BPM). The paired difference was significant (two-sided Wilcoxon signed-rank test, W = 131, *p* = 3.0 × 10^−4^).

On PURE, soft-argmax achieved a lower MAE than GAP in 8 of 10 subjects. The median subject-level MAE was 3.09 BPM for GAP and 2.32 BPM for soft-argmax, with a median paired reduction of 0.82 BPM (95% bootstrap CI, 0.22–2.85 BPM). The paired difference was also significant (W = 3, *p* = 9.8 × 10^−3^). However, the large segment-pooled difference on PURE was amplified by one high HR subject. For this subject, the MAE was 50.12 BPM with GAP and 9.12 BPM with soft-argmax. After excluding this subject in a post hoc diagnostic analysis, the segment-pooled MAEs were 3.36 BPM for GAP and 2.29 BPM for soft-argmax. Soft-argmax remained better in seven of the remaining nine subjects, but this restricted analysis should be interpreted cautiously because it was post hoc and included only nine subjects.

These findings support an effect of readout design independent of the shared convolutional backbone and the shared local Conv1D feature extractor. The typical subject-level improvement was modest, with median paired reductions of approximately 0.8 BPM, whereas the benefit was substantially larger for the high HR PURE subject. The matched comparison therefore does not indicate that GAP failed uniformly. Instead, GAP retained sufficient implicit frequency information to provide reasonable estimates for many subjects, but the structured soft-argmax readout was more accurate for most subjects.

To further test whether the shared local Conv1D processing accounted for the observed difference, we repeated the PURE LOSO evaluation after removing this block from GAP, bounded GAP, and soft-argmax. The resulting segment-pooled MAEs were 7.54, 7.83, and 2.79 BPM, respectively, compared with 8.09, 8.53, and 2.98 BPM for the corresponding models with the shared Conv1D block. The relative ordering was therefore unchanged. Moreover, soft-argmax without the shared Conv1D still outperformed GAP with Conv1D at the subject level (median paired MAE reduction, 0.86 BPM; 95% bootstrap CI, 0.23–2.57 BPM; W = 5, *p* = 0.0195). The proposed model also contained slightly fewer trainable parameters (119,921) than the matched GAP and bounded-GAP controls (122,001 each). Together, these controls indicate that the observed advantage cannot be attributed simply to the presence of the shared local Conv1D feature extractor or to a larger parameter count. This inverse ablation also provides a direct test of the readout after removal of the shared post-fusion local feature-extraction block.

GMP exhibited a different failure pattern. It retained only the maximum activation in each feature channel and discarded both its frequency position and the remaining activation distribution. Its segment-pooled MAE was 22.30 BPM on PURE and 22.20 BPM on UBFC-rPPG. Although performance varied across subjects, the overall results were poor on both datasets.

The dense regression head produced MAEs of 10.06 BPM on PURE and 5.42 BPM on UBFC-rPPG when all test segments were pooled. As observed with GAP, the PURE result was strongly influenced by the subject with high HR values. On UBFC-rPPG, dense regression remained less accurate than soft-argmax, with MAEs of 5.42 and 3.82 BPM, respectively. Although flattening preserved the ordering of the frequency-indexed features and therefore allowed frequency position to be learned implicitly, the head did not explicitly map its output to predefined BPM coordinates. These results suggest that retaining the frequency dimension in the backbone was not sufficient to reproduce the performance of the structured soft-argmax readout.

Soft-argmax differs from GAP in more than one respect. It retains an explicit BPM-position axis, produces a normalized distribution over frequency bins, calculates a coordinate-based expectation, and constrains the output to the predefined range of 45–150 BPM. We therefore evaluated a bounded GAP control to determine whether matching the output range alone could reproduce the soft-argmax behavior. The controls reported here progressively address the shared local Conv1D block, output-range restriction, and model capacity; they do not independently separate probabilistic normalization from coordinate-based expectation.

#### 4.2.2. Bounded-Output Control

To determine whether output range restriction alone accounted for the improvement, we implemented a bounded GAP control using the same frequency-preserving backbone as the unbounded GAP model. Its scalar output was constrained to the same 45–150 BPM range as soft-argmax. All three readouts were evaluated using the complete LOSO protocol on PURE and UBFC-rPPG.

On UBFC-rPPG, bounded GAP reduced the segment-pooled MAE from 5.14 BPM for unbounded GAP to 4.19 BPM (Table 3), while soft-argmax achieved 3.82 BPM in the primary run. To assess random-seed sensitivity, we repeated the complete 38-fold LOSO comparison of soft-argmax and bounded GAP with seeds 42, 43, and 44 under matched code, device, splits, optimization, and early-stopping conditions. Pooled MAE was 3.69/3.84/3.98 BPM for soft-argmax and 4.27/4.50/4.27 BPM for bounded GAP (mean ± SD, 3.83 ± 0.14 vs. 4.35 ± 0.13 BPM). Subject-macro-MAE (4.36 ± 0.15 vs. 4.79 ± 0.14 BPM) and within ±5 BPM (78.9 ± 0.6% vs. 73.5 ± 0.7%) also favored soft-argmax in all three seeds. RMSE and Pearson’s r reversed in seed 44. Soft-argmax had lower subject-level MAE in 27, 27, and 28 of 38 subjects, with median paired reductions of 0.442, 0.491, and 0.277 BPM and Wilcoxon *p* values of 0.0076, 0.0012, and 0.0719. Thus, the MAE-based advantage was directionally consistent across seeds, whereas effect magnitude and statistical significance were seed-dependent.

On PURE, bounded GAP achieved a segment-pooled MAE of 8.53 BPM, compared with 8.09 BPM for unbounded GAP and 2.98 BPM for soft-argmax. In a post hoc diagnostic analysis of the remaining nine subjects after excluding the high HR subject, bounded GAP reduced the MAE from 3.36 to 2.93 BPM but remained less accurate than soft-argmax at 2.29 BPM.

For the high HR subject, bounded GAP produced predictions of only 56–87 BPM despite allowing outputs up to 150 BPM. Its MAE was 58.26 BPM, compared with 50.12 BPM for unbounded GAP and 9.12 BPM for soft-argmax. Thus, matching the allowable output range did not prevent the scalar regression failure in this subject.

These results show that output range restriction alone was insufficient to explain the soft-argmax result on PURE and did not fully reproduce it on UBFC-rPPG. Thus, output range restriction contributed to performance on UBFC-rPPG, but the remaining difference should be interpreted as an effect of the structured readout rather than of frequency localization alone.

#### 4.2.3. Comparison of CHROM and POS Inputs

We examined whether the performance of the proposed architecture depended on the method used to extract the pulse-like input signal. CHROM was replaced with POS while the frequency-preserving soft-argmax network, Morlet wavelet, and time–frequency grid were held fixed. Replacing CHROM with POS increased the MAE by only 0.01 BPM on PURE and 0.15 BPM on UBFC-rPPG (Table 4). These small differences suggest that the observed performance was not specific to the CHROM input under the evaluated conditions.

#### 4.2.4. Results of the Frequency-Axis Resolution Sensitivity Analysis

Reducing the frequency axis from 300 to 100 bins produced very similar performance. In the internally matched reruns, the segment-pooled MAEs for 300, 100, and 50 bins were 2.76, 2.82, and 3.33 BPM on PURE and 3.92, 4.00, and 3.87 BPM on UBFC-rPPG, respectively. On PURE, the corresponding RMSE values were 5.63, 5.08, and 7.34 BPM, with Pearson correlations of 0.971, 0.978, and 0.952 and within ±5 BPM rates of 88.1%, 87.0%, and 86.4%. On UBFC-rPPG, the RMSE values were 6.91, 6.95, and 6.56 BPM, correlations were 0.907, 0.905, and 0.917, and within ±5 BPM rates were 79.4%, 77.8%, and 78.4%.

At the subject level, the 300- and 100-bin variants did not differ significantly on either dataset (PURE: W = 18, *p* = 0.375; UBFC-rPPG: W = 312, *p* = 0.405). Reducing the axis further to 50 bins significantly degraded MAE on PURE (W = 7, *p* = 0.037) but not on UBFC-rPPG (W = 354, *p* = 0.819). On PURE, most of the 50-bin degradation was concentrated in the out-of-range subject examined in Section 4.3.1: its MAE increased from 7.59 BPM at 300 bins to 12.12 BPM at 50 bins, whereas the remaining nine subjects changed by at most 0.37 BPM.

These results show that retaining all 300 sampled frequency coordinates is not strictly necessary, because 100 bins yielded statistically comparable subject-level performance on both primary datasets. Denser frequency-coordinate sampling was more robust on PURE, particularly for the out-of-range subject, but this tendency was not consistent on UBFC-rPPG. Accordingly, the role of the architecture is better described as preserving an explicit frequency-coordinate representation through the readout rather than requiring the original 300-bin sampling density itself.

#### 4.2.5. Cross-Attention Fusion Control

We next compared element-wise averaging with cross-attention while holding the backbone, soft-argmax readout, preprocessing, training protocol, and LOSO splits fixed. On PURE, element-wise averaging achieved an MAE of 2.76 BPM, RMSE of 5.63 BPM, Pearson’s r of 0.971, and a within ±5 BPM rate of 88.1%, compared with 3.06 BPM, 6.67 BPM, 0.960, and 87.3%, respectively, for cross-attention. On UBFC-rPPG, the corresponding values were 3.92 BPM, 6.91 BPM, 0.907, and 79.4% for averaging versus 4.23 BPM, 7.31 BPM, 0.896, and 76.3% for cross-attention.

At the subject level, the MAE differences were not statistically significant on either dataset (PURE: *p* = 0.131; UBFC-rPPG: *p* = 0.084). Cross-attention yielded lower MAE for 3 of 10 PURE subjects, with each improvement smaller than 0.35 BPM, and for 14 of 38 UBFC-rPPG subjects. The improvements were therefore not sufficiently consistent to alter the overall ranking. Cross-attention also increased the number of trainable parameters from 119,921 to 170,609 without providing a measurable accuracy benefit. Accordingly, element-wise averaging was retained as the primary fusion strategy.

The learned pairwise cross-attention showed little evidence of strongly selective cross-ROI frequency interactions. Because frequency bins rather than ROIs themselves were used as attention tokens, the normalized attention entropy characterized the selectivity of frequency-to-frequency attention across ROI pairs rather than direct ROI-level weighting. Normalized attention entropy was 0.982 on PURE and 0.985 on UBFC-rPPG, close to the uniform value of 1.0. Correlations between attention statistics and the evaluated input-quality measures were weak (|ρ| = 0.04–0.19). Cross-attention also showed no consistent advantage across the controlled PURE motion conditions; for example, in the Talking condition, the MAE was 3.36 BPM with element-wise averaging and 3.54 BPM with cross-attention. Thus, within the present architecture, the implemented cross-attention did not provide evidence that selective cross-ROI frequency interactions improved HR estimation.

### 4.3. Residual Error and Input Signal Quality Analysis

Section 4.2 showed that the structured frequency-preserving readout reduced estimation error relative to the matched backbone GAP readout but did not eliminate all errors. We therefore examined two prominent failure patterns: a high HR case on PURE and subject-specific input-signal failures on UBFC-rPPG. These analyses were conducted to determine whether the remaining errors were attributable primarily to the learned readout or were already reflected in the extracted camera signals.

#### 4.3.1. HR-Range Mismatch on PURE

The largest error on PURE occurred for subject sub07. Across all 5890 analyzed PURE segments, the reference HR ranged from 44.0 to 143.6 BPM. A total of 582 segments (9.88%) had reference HR values of at least 120 BPM, and all 582 originated from sub07. When sub07 was held out in LOSO evaluation, the remaining training subjects covered only 44.0–103.8 BPM and contained no segments above 110 BPM, whereas the held-out subject covered 118.1–143.6 BPM. Thus, the entire sub07 test range lay above the empirically observed upper HR range of its training fold. Importantly, the proposed soft-argmax readout is structurally bounded to the predefined 45–150 BPM output range. Because sub07 reaches 143.6 BPM, the highest-HR examples lie close to this upper bound, leaving only a small output margin and requiring particular caution when interpreting performance in this range. In contrast, UBFC-rPPG contained 3386 analyzed segments spanning 58.3–124.6 BPM, of which only 108 (3.19%) reached at least 120 BPM and these came from three subjects. For sub07, the proposed soft-argmax model produced an MAE of 9.12 BPM, whereas matched GAP, bounded GAP, and dense regression produced MAEs of 50.12, 58.26, and 67.68 BPM, respectively. The classical CHROM-FFT and POS-FFT estimators achieved approximately 4.4 and 4.0 BPM, respectively.

**Figure 4:**
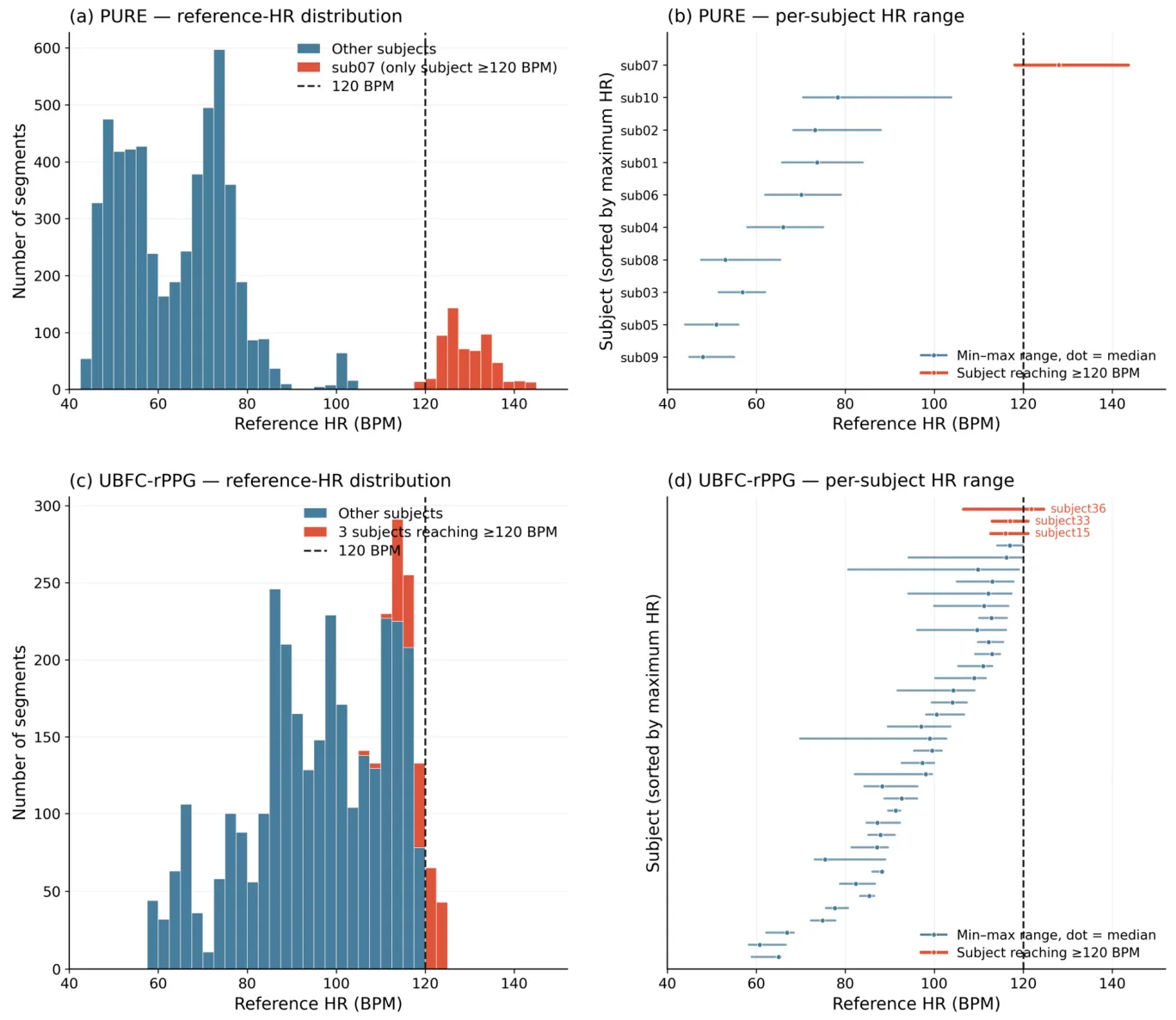
Reference heart-rate distributions in the two primary datasets. (**a**,**c**) Histograms of reference HR over all analyzed segments, with segments from subjects reaching ≥120 BPM highlighted separately (PURE: *n* = 5890 from 10 subjects, 44.0–143.6 BPM; UBFC-rPPG: *n* = 3386 from 38 subjects, 58.3–124.6 BPM). (**b**,**d**) Per-subject HR ranges (minimum–maximum; dots indicate medians), sorted by maximum HR. In PURE, all 582 segments with reference HR ≥ 120 BPM originated from a single subject (sub07). When this subject was held out in LOSO evaluation, the remaining training subjects contained no segments above 110 BPM. In UBFC-rPPG, only three subjects reached 120 BPM, and the maximum reference HR was 124.6 BPM.

Band-wise analysis further localized the degradation to sparsely represented or unobserved HR regions. Complete HR-stratified results, including the number of test segments, MAE, RMSE, and the proportion of estimates within ±5 BPM for each method, are reported in Appendix A (Table A1 and Table A2). In PURE, the numbers of segments in the <60, 60–80, 80–100, 100–120, 120–140, and ≥140 BPM bands were 2363, 2615, 236, 94, 554, and 28, respectively. In the 120–140 BPM band, the proposed soft-argmax readout achieved an MAE of 8.29 BPM, an RMSE of 15.55 BPM, and a within ±5 BPM rate of 58.5%. This remained substantially better than matched GAP (49.60 BPM, 50.43 BPM, 0.0%) and bounded GAP (58.08 BPM, 58.71 BPM, 0.0%), but the classical CHROM-FFT and POS-FFT estimators were more accurate (MAE 4.11 and 4.00 BPM; within ±5 BPM 86.1% and 86.5%, respectively). At ≥140 BPM, the proposed method degraded further to an MAE of 29.19 BPM, an RMSE of 31.13 BPM, and a within ±5 BPM rate of 0.0%, confirming that the structured readout reduced but did not eliminate the extreme out-of-range failure. For UBFC-rPPG, the corresponding band counts were 44, 494, 1282, 1458, and 108 up to the 120–140 BPM band. The proposed model achieved its lowest MAEs in the 100–120 and 120–140 BPM bands (2.72 and 2.38 BPM, respectively; within ±5 BPM, 85.8% and 88.0%), whereas performance was poorer below 80 BPM (8.17 BPM for <60 BPM and 6.94 BPM for 60–80 BPM). These results indicate that the observed degradation is associated with sparse or out-of-distribution HR coverage rather than with high HR per se, although training-range coverage is not established as the sole cause. The corresponding dataset-level HR histograms and per-subject HR ranges are shown in Figure 4.

Conventional oversampling could not be applied to the critical sub07 fold because no genuine high-HR training segments were available to resample. In the exploratory synthetic frequency-axis augmentation experiment, soft-argmax showed no consistent improvement across three seeds: the sub07 MAE was 6.80 ± 1.18 BPM without augmentation and 9.26 ± 4.81 BPM with augmentation, with the direction of the effect varying across seeds. For matched GAP, augmentation reduced MAE from 45.67 ± 6.40 to 33.91 ± 2.28 BPM, but the within ±5 BPM rate remained 0% in both conditions. Thus, synthetic frequency-axis augmentation did not reliably resolve the high-HR failure, and validation using genuine training subjects that cover the missing HR range remains necessary.

**Figure 5:**
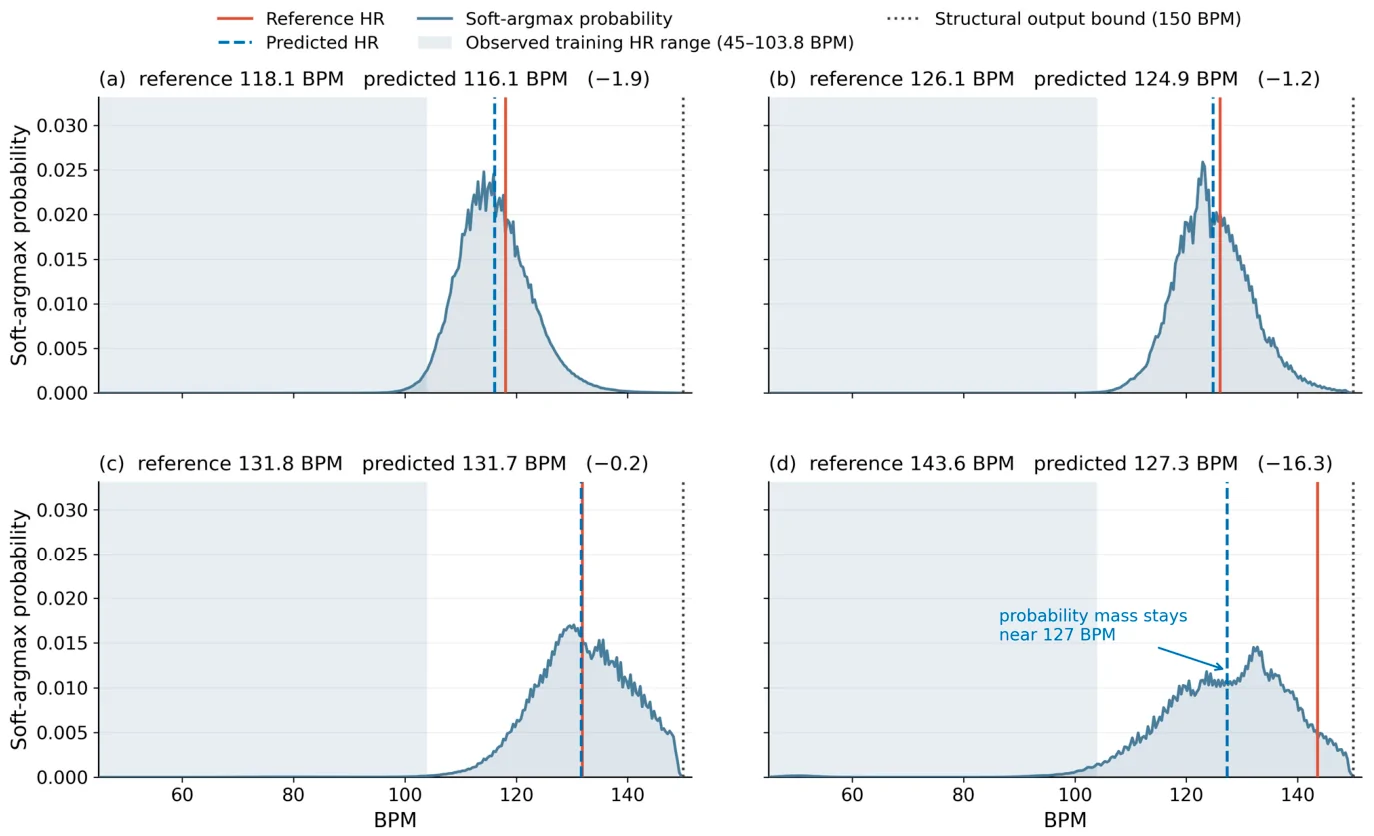
Representative soft-argmax probability profiles for the high-HR PURE subject (sub07). Panels show segments with reference/predicted HR values of (**a**) 118.1/116.1 BPM, (**b**) 126.1/124.9 BPM, (**c**) 131.8/131.7 BPM, and (**d**) 143.6/127.3 BPM. Solid red and dashed blue lines indicate the reference and predicted HR, respectively. The shaded region denotes the observed HR range of the training subjects in the sub07 LOSO fold (44.0–103.8 BPM), while the dotted line marks the structural upper output bound of the soft-argmax readout (150 BPM). Although the model extrapolated beyond the observed training range in panels (**a**–**c**), the probability mass remained concentrated near 127 BPM for the highest-HR example, resulting in substantial underestimation in panel (**d**).

Figure 5 shows representative soft-argmax BPM probability profiles for sub07. The model localized HR accurately for examples between approximately 118 and 132 BPM despite these values lying above the observed training range. However, for the example with a reference HR of 143.6 BPM, the predicted probability mass remained concentrated near 127 BPM, resulting in substantial underestimation. This example is also close to the structural upper output bound of 150 BPM. This pattern is consistent with the limited upper-range training coverage, while the augmentation results above indicate that frequency-range manipulation alone is insufficient to establish or correct the underlying cause.

#### 4.3.2. Subject-Specific Input-Signal Failure Patterns on UBFC-rPPG

The three subjects with the largest errors for the proposed model were subject26, subject25, and subject27, with subject-level MAEs of 23.9, 20.3, and 17.1 BPM, respectively. To investigate whether these errors were associated with the reference labels, color signal extraction, or facial motion, we compared the contact PPG, individual RGB channels, CHROM and POS signals, ICA components, and ROI-centroid motion for each subject.

We first examined the reference signals. Spectral analysis of the contact PPG produced dominant HR values of approximately 78 BPM for subject26, 90 BPM for subject25, and 87 BPM for subject27, consistent with the supplied reference labels. These findings provide no indication that incorrect reference labels were the primary source of the large errors. Moreover, the erroneous peaks were not located at simple integer multiples or submultiples of the reference HR, making conventional harmonic or subharmonic confusion an unlikely common explanation.

For subject26, the median segment-level dominant frequencies of both CHROM and POS were approximately 123 BPM, whereas the green channel showed a median dominant frequency near 82 BPM, closer to the reference value of approximately 78 BPM. ROI-centroid displacement was small across the three ROIs, ranging from approximately 0.7 to 1.0 pixels, and little motion power was observed near the erroneous frequency. The substantial difference between the projected signals and the green channel suggests that the color-projection step altered the dominant frequency and may have amplified the estimation error in this subject. We therefore describe this case as a projection-sensitive failure pattern. Nevertheless, the green channel estimate was within 10 BPM of the reference in only 37% of the segments. Thus, using the green channel alone reduced but did not eliminate the error.

For subject25, CHROM, POS, the green channel, and ICA all showed a dominant frequency near 144 BPM rather than the reference value of approximately 90 BPM. Because this erroneous component remained present across multiple RGB-derived signal representations, it could not be attributed specifically to CHROM. ROI-centroid displacement was also small across the three ROIs, ranging from approximately 0.4 to 0.5 pixels, with little motion power near 144 BPM. We use the term projection-persistent RGB domain failure pattern to denote this persistence of the erroneous periodic component across multiple signal-extraction methods despite little observable rigid motion.

Subject27 exhibited a different pattern. ROI-centroid displacement was substantially larger across the three ROIs, ranging from approximately 6.2 to 6.7 pixels, and the motion signal contained power near the frequencies associated with the estimation errors. In addition, different color projection methods produced different erroneous dominant frequencies. These observations are consistent with motion-related contamination of the extracted color signals, and we therefore describe this case as a motion-associated failure pattern. However, ROI-centroid motion does not capture all forms of non-rigid facial deformation.

Small ROI-centroid displacement does not exclude non-rigid facial motion, local deformation, or illumination-related interference. Accordingly, the physical causes of the errors in subject25 and subject26 could not be determined from the available measurements. The terms projection-sensitive, projection-persistent, and motion-associated are therefore used only to summarize the observed signal patterns and should not be interpreted as statistically established failure mechanisms.

Despite their different case-specific patterns, subject25, subject26, and subject27 also showed large errors with CHROM-FFT and POS-FFT. The occurrence of high errors across both learned and non-learned estimators indicates that these failures were not unique to the proposed readout. Instead, the results support the interpretation that prominent non-cardiac components were already present in the camera-derived signals before application of the learned readout.

#### 4.3.3. Reference-Free Quality Score with Fold-Wise Calibration

After correcting the quality-score calibration to use training subjects only within each LOSO fold, the numerical results changed negligibly. At the 15% rejection condition used in this exploratory analysis, the pooled UBFC-rPPG MAE decreased from 3.82 BPM before rejection to 3.13 BPM after rejection; the corresponding value with the original full-cohort calibration had been 3.14 BPM. The association between Q and absolute HR error was essentially unchanged after fold-wise calibration. These findings indicate that the previously reported numerical behavior was not driven by the calibration leakage. However, because the feature combination and rejection proportion were selected post hoc and no independent external dataset was used for validation, the quality score should be interpreted only as an exploratory indicator of input reliability rather than as a deployment-ready rejection rule.

### 4.4. Controlled Motion and Illumination Analyses

We examined estimation errors under the controlled motion conditions in PURE and the controlled illumination conditions in BH-rPPG. The PURE analysis was used to describe differences among motion types, whereas the BH-rPPG analysis tested whether reduced illumination affected the evaluated estimators differently.

#### 4.4.1. Motion Conditions on PURE

PURE contains six recording conditions: Steady, Talking, Slow Translation, Fast Translation, Small Rotation, and Medium Rotation. Because the high HR subject examined in Section 4.3.1 produced substantially larger errors than the other subjects, we conducted a post hoc diagnostic analysis after excluding this subject. The primary within-dataset results reported in Section 4.1 include all subjects.

After exclusion of the high HR subject, the proposed method achieved MAEs of 1.87–2.32 BPM under the four translation and rotation conditions, similar to the Steady-condition MAE of 1.99 BPM (Table 5). The Talking condition showed a higher MAE of 3.50 BPM and a lower within ±5 BPM rate of 73.3%. When the high HR subject was included, the Talking-condition MAE was 6.57 BPM.

These results indicate that the proposed method maintained similar accuracy under the evaluated rigid translation and rotation conditions, whereas Talking was associated with larger errors. Talking involves non-rigid facial movements around the mouth and cheeks in addition to possible head motion, which may affect the spatially averaged color signals used by CHROM.

#### 4.4.2. Illumination Conditions on BH-rPPG

BH-rPPG contains 35 subjects recorded under low, medium, and high illumination conditions. We applied the same CHROM preprocessing, CWT settings, 10 s analysis window, and 0.5 s stride used in the PURE and UBFC-rPPG experiments. Evaluation was performed using 35-fold LOSO cross-validation. In each fold, all three illumination conditions from one subject were held out together for testing.

We evaluated illumination effects at two aggregation levels. Table 6 reports MAEs calculated by pooling all test segments within each illumination condition. Statistical comparisons were instead conducted at the subject level. For each subject, illumination-induced degradation was defined as the MAE under low illumination minus the MAE under high illumination, yielding one degradation value per subject (*n* = 35).

All four methods showed higher errors as illumination decreased. In the segment-pooled analysis, the proposed method increased from 5.85 BPM under high illumination to 9.30 BPM under low illumination, corresponding to an increase of 3.45 BPM. The increases were 4.08 BPM for CWT-argmax, 6.69 BPM for CHROM-FFT, and 7.86 BPM for POS-FFT. The proposed method achieved the lowest MAE under low illumination, whereas POS-FFT achieved the lowest MAEs under medium and high illumination. Thus, the proposed method was not uniformly the most accurate across all illumination conditions; its advantage was a smaller deterioration as illumination decreased.

The subject-level analysis showed the same pattern. The mean high-to-low degradation was 3.52 BPM for the proposed method, 4.10 BPM for CWT-argmax, 6.70 BPM for CHROM-FFT, and 7.82 BPM for POS-FFT. These values differ slightly from the segment-pooled differences because the subject-level analysis assigns equal weight to each subject.

In the three planned paired comparisons, the proposed method showed significantly less degradation than both FFT-based estimators. After Holm’s correction, the differences remained significant relative to CHROM-FFT (Wilcoxon *p* = 0.036; permutation *p* = 0.030) and POS-FFT (Wilcoxon *p* = 0.015; permutation *p* = 0.018). In contrast, no significant difference in degradation was detected between the proposed method and CWT-argmax (Wilcoxon *p* = 0.565; permutation *p* = 0.640).

These results show that the proposed CWT-based model was less affected by reduced illumination than the two FFT-based estimators. The non-learned CWT-argmax baseline showed a similarly small degradation, suggesting that the observed illumination robustness was associated with properties shared by the CWT-based estimation approaches rather than being attributable specifically to the learned soft-argmax readout. However, the current comparisons do not determine whether this pattern resulted from the time–frequency representation itself, the explicit localization of frequency, or their combination. Moreover, CWT-based estimation was not immune to reduced illumination; as shown in Section 4.4.3, the cardiac-frequency structure of the input scalograms also deteriorated under low illumination.

#### 4.4.3. Illumination-Related Changes in the Input Representation

Because estimation error increased as illumination decreased in Section 4.4.2, we next examined whether this degradation was already detectable in the CHROM-derived signals and CWT scalograms before application of the learned readout. The reference-dependent non-cardiac dominance score increased from 19.7% under high illumination to 24.4% under medium illumination and 45.4% under low illumination. Thus, as illumination decreased, a larger proportion of segments contained a dominant CHROM frequency that differed from the reference HR by more than 10 BPM.

**Figure 6:**
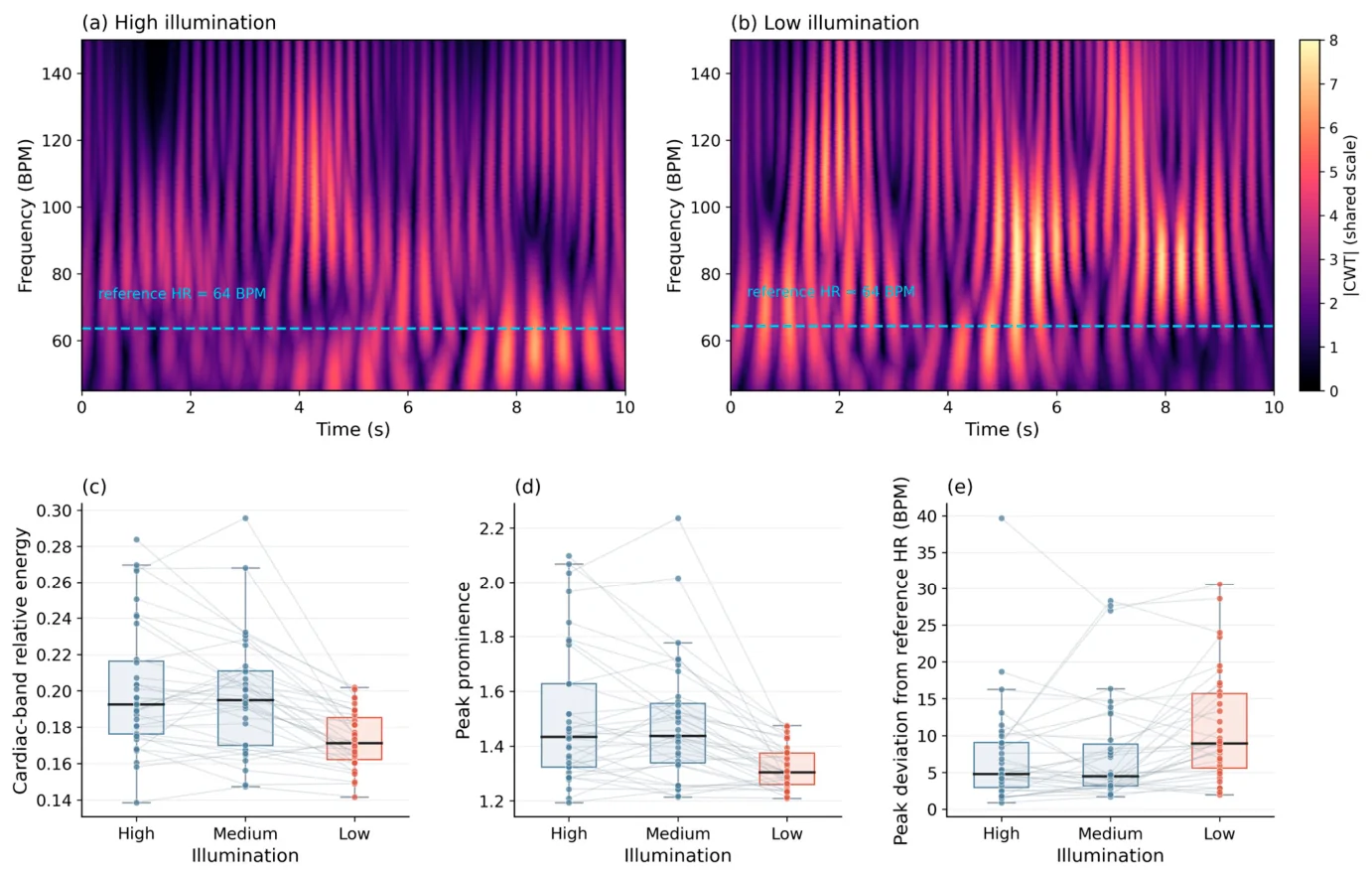
Illumination-related changes in the CWT representation on BH-rPPG. (**a**,**b**) Representative CWT scalograms from the same subject under high and low illumination, displayed using a common color scale. The dashed cyan line indicates the reference HR. (**c**–**e**) Subject-level changes across high, medium, and low illumination in cardiac-band relative energy, peak prominence, and absolute deviation of the dominant CWT peak from the reference HR, respectively. Each connected line represents the same subject across illumination conditions; boxes indicate the median and interquartile range. With decreasing illumination, cardiac-band energy and peak prominence decreased, whereas the deviation of the dominant CWT peak from the reference HR increased, indicating that the cardiac-frequency structure became weaker and less clearly localized before application of the learned readout.

The CWT scalograms showed corresponding changes. From high to low illumination, cardiac band relative energy decreased from 0.202 to 0.173, peak prominence decreased from 1.51 to 1.32, and the absolute deviation of the dominant CWT peak from the reference HR increased from 7.04 to 11.16 BPM. These changes indicate that the cardiac-related spectral component became weaker and less clearly localized under low illumination before application of the learned readout, as illustrated by the representative scalograms in Figure 6a,b and the subject-level summaries in Figure 6c–e.

These results show that illumination-related degradation was already detectable in the CHROM-derived signals and CWT scalograms before application of the learned readout. Under lower illumination, the cardiac-related component became weaker, less prominent, and less accurately localized in frequency. Nevertheless, as shown in Section 4.4.2, the proposed model achieved the lowest segment-pooled MAE under low illumination, and both CWT-based estimators showed smaller high-to-low degradation than the evaluated FFT-based estimators. This pattern is consistent with the CWT retaining time-localized frequency structure even when the cardiac component is weakened. However, the CWT does not inherently remove illumination noise, and the present analysis does not isolate time–frequency localization as the sole cause of the observed robustness. Because the degradation of the proposed model did not differ significantly from that of CWT-argmax, the relative robustness also cannot be attributed specifically to the learned soft-argmax readout.

### 4.5. Agreement and Cross-Dataset Transfer

We further examined the agreement between the estimated and reference HR values and evaluated the transfer of the proposed model between PURE and UBFC-rPPG.

#### 4.5.1. Descriptive Agreement Analysis

**Figure 7:**
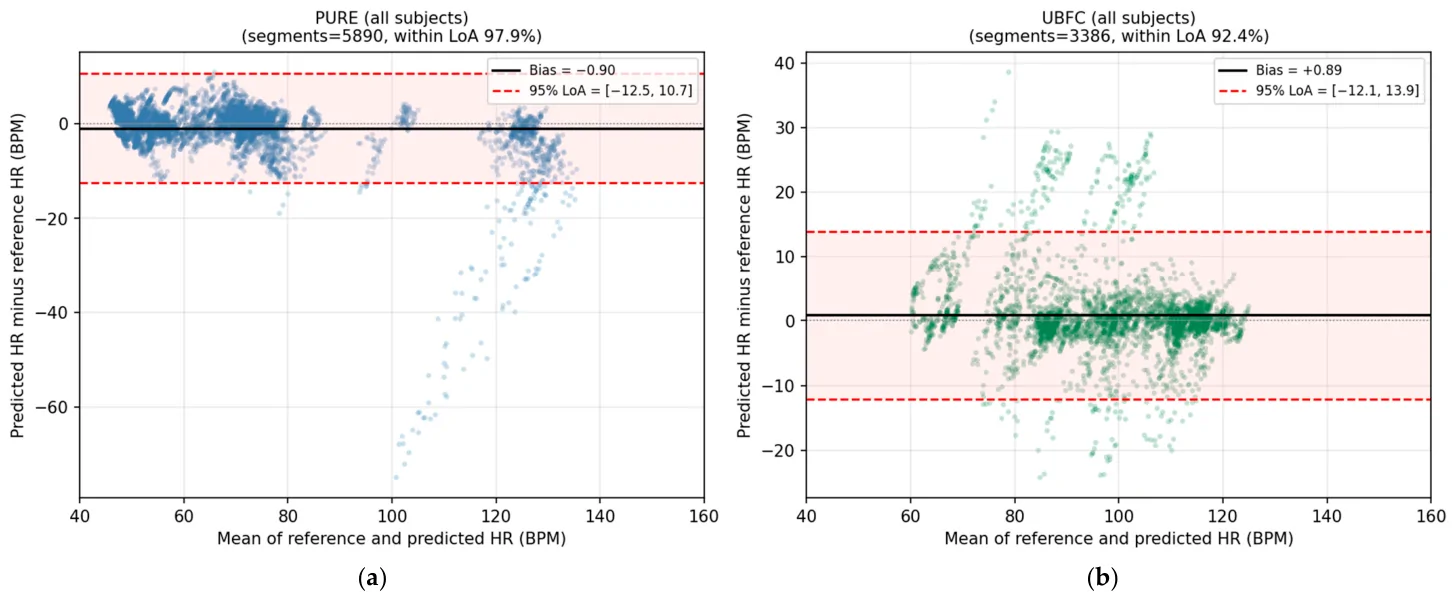
Descriptive segment-level Bland–Altman plots for the proposed model under LOSO cross-validation: (**a**) PURE and (**b**) UBFC-rPPG. The difference was calculated as predicted HR minus reference HR. Solid black lines indicate the mean bias, and red dashed lines indicate the descriptive limits of agreement, calculated as the mean difference ± 1.96 SD. Because the 10 s segments overlapped and multiple observations were obtained from each subject, the plots are presented as descriptive summaries and were not used for population-level inference.

Figure 7 shows segment-level Bland–Altman plots for PURE and UBFC-rPPG. The difference was calculated as estimated HR minus reference HR. Because consecutive 10 s segments overlapped and multiple segments were obtained from each subject, the observations were not statistically independent. The plots and the corresponding mean ± 1.96 SD limits are therefore presented as descriptive summaries of the observed error distributions rather than as population-level limits of agreement.

When all subjects were included, the mean bias was −0.90 BPM on PURE and +0.89 BPM on UBFC-rPPG. The corresponding empirical limits were [−12.5, +10.7] BPM for PURE and [−12.1, +13.9] BPM for UBFC-rPPG. Although the overall biases were small, they should not be interpreted as uniformly close agreement because substantial errors remained in specific subjects and HR ranges.

We also calculated agreement statistics after excluding the failure cases examined in Section 4.3 as a post hoc diagnostic analysis. After excluding the high HR PURE subject, the mean bias changed from −0.90 to −0.02 BPM, and the empirical limits narrowed from [−12.5, +10.7] BPM to [−6.1, +6.0] BPM. After excluding the three high-error UBFC-rPPG subjects, the mean bias changed from +0.89 to −0.16 BPM, and the empirical limits narrowed from [−12.1, +13.9] BPM to [−9.5, +9.1] BPM.

These diagnostic exclusions reduced both the overall bias and the spread of the errors, indicating that a small number of subject-specific failure cases had a substantial influence on the segment-level agreement statistics.

#### 4.5.2. Cross-Dataset Transfer

We next evaluated cross-dataset transfer without fine-tuning. The model was trained using one primary dataset and directly evaluated on the other dataset without fine-tuning, using the same preprocessing and input construction.

Transfer performance differed substantially between the two directions (Table 7). When trained on PURE and tested on UBFC-rPPG, the proposed model achieved an MAE of 4.08 BPM and a Pearson correlation coefficient of r = 0.92. In a post hoc diagnostic analysis excluding the three high-error UBFC-rPPG subjects examined in Section 4.3.2, the MAE decreased to 3.06 BPM. This reduction indicates that the subject-specific input-signal failures identified in the within-dataset analysis also affected cross-dataset transfer.

In the reverse direction, training on UBFC-rPPG and testing on PURE produced an MAE of 8.44 BPM, although the Pearson correlation remained high. The UBFC-rPPG training pipeline had already applied a 50–130 BPM reference-HR restriction; within the retained 38-subject cohort, the actual observed HR range was 58.3–124.6 BPM. Thus, reapplying the 50–130 BPM restriction would not define a new retraining condition. We therefore stratified the PURE test segments according to this empirically observed training range. The MAE was 2.61 BPM for the 3155 segments within 58.3–124.6 BPM, compared with 16.72 BPM for the 2250 segments below this range and 7.88 BPM for the 485 segments above it. Accordingly, 38.2% of PURE segments fell below the UBFC-rPPG training range, whereas 8.2% fell above it. These findings indicate that HR-range mismatch, particularly on the low-HR side, was an important contributor to the transfer error. However, because the stratification was defined post hoc using test reference HR, it does not establish HR-range mismatch as the sole cause; differences in camera characteristics, illumination, motion patterns, subject populations, and other dataset-specific factors may also have contributed.

The restricted analyses in both transfer directions rely on information identified after examining the test data: subject identity in PURE-to-UBFC-rPPG transfer and reference HR in UBFC-rPPG-to-PURE transfer. They are therefore diagnostic decompositions rather than deployable selection procedures or alternative generalization results. The all-segment MAEs of 4.08 and 8.44 BPM remain the primary cross-dataset results.

Overall, the transfer findings show that the proposed model retained useful predictive ability across datasets but did not generalize uniformly in both directions. The post hoc stratification indicates that mismatch in the physiological HR range represented during training was an important contributor, while dataset-specific differences in acquisition conditions and input-signal quality likely also affected transfer performance.

### 4.6. Computational Cost

The proposed model contained 119,921 trainable parameters. On an Intel Core i7-12700H laptop CPU, the network forward pass required 34.61 ± 3.88 ms per 10 s segment at a batch size of 1. CHROM extraction and CWT scalogram generation required 13.78 ± 0.71 ms and 15.55 ± 1.44 ms per ROI, respectively. Including the measured preprocessing stages for the three ROIs, the estimated total processing time was approximately 122.7 ms per segment. Because a new segment was generated every 0.5 s in the present evaluation, these measured stages were compatible with near-real-time processing on the evaluated laptop CPU. The timing estimate excludes video acquisition and MediaPipe FaceMesh landmark detection and therefore should not be interpreted as fully end-to-end system latency.

## 5. Discussion

### 5.1. Main Finding

The main finding of this study is that the complete structured soft-argmax readout improved HR estimation from CWT scalograms relative to the evaluated GAP-based readouts within the shared frequency-preserving architecture. In a CWT scalogram, the vertical position represents frequency and is therefore directly related to HR. GAP does not retain this frequency coordinate explicitly after aggregation, although preceding convolutional layers may encode some position-related information implicitly in their feature channels. The present ablations make shared local Conv1D processing, output-range restriction, and model capacity less likely alternative explanations, but probabilistic normalization and coordinate-based expectation remain coupled. The results therefore support the complete structured soft-argmax formulation within the evaluated CHROM-CWT pipeline, rather than establishing that frequency-coordinate preservation alone is sufficient to explain the observed gain.

The conventional GAP-based CWT-CNN was retained as a broader baseline using the same CHROM-CWT inputs but a different convolutional backbone and readout design. Because this comparison changes more than one architectural component, it cannot isolate the source of any performance difference. We therefore focus the central architectural interpretation on the matched-backbone ablation, in which the CHROM-CWT input, frequency-preserving backbone, ROI fusion, training protocol, and LOSO splits were held fixed and the readout mechanism was changed.

In this matched comparison, replacing soft-argmax with GAP increased the segment-pooled MAE from 2.98 to 8.09 BPM on PURE and from 3.82 to 5.14 BPM on UBFC-rPPG. The difference was also consistent at the subject level. Soft-argmax achieved lower MAE in 8 of 10 PURE subjects and 31 of 38 UBFC-rPPG subjects. The paired differences were significant on both PURE (two-sided Wilcoxon signed-rank test, W = 3, *p* = 9.8 × 10^−3^) and UBFC-rPPG (W = 131, *p* = 3.0 × 10^−4^), with median paired reductions of 0.82 and 0.85 BPM, respectively. Importantly, the matched GAP controls already used the same post-fusion Conv1D (32, kernel size 5) feature extractor as soft-argmax, so the comparison did not differ simply by the presence of local Conv1D processing.

The inverse Conv1D ablation provided a complementary control. Removing the shared post-fusion Conv1D feature extractor from all three readouts yielded pooled PURE MAEs of 7.54 BPM for GAP, 7.83 BPM for bounded GAP, and 2.79 BPM for soft-argmax, preserving the same ordering observed with the shared Conv1D block. Soft-argmax without this block also remained significantly better than GAP with the block at the subject level (median paired reduction, 0.86 BPM; 95% CI, 0.23–2.57 BPM; *p* = 0.0195). In addition, the proposed model used 119,921 trainable parameters, slightly fewer than the 122,001 parameters in the matched GAP controls. These findings make local Conv1D processing and parameter count unlikely explanations for the advantage, although they do not independently isolate probabilistic normalization from coordinate-based expectation.

The magnitude of this benefit was not uniform across datasets, subjects, or random initializations. On PURE, the pooled difference was amplified by one high-HR subject. On UBFC-rPPG, the added three-seed sensitivity analysis preserved the pooled- and subject-macro-MAE ordering in every seed (pooled MAE, 3.83 ± 0.14 BPM for soft-argmax vs. 4.35 ± 0.13 BPM for bounded GAP). However, the subject-level Wilcoxon *p* values ranged from 0.0012 to 0.0719. The additional benefit over bounded GAP should therefore be interpreted based on the consistency of the MAE-based ordering across seeds rather than on statistical significance in any single initialization, and the remaining difference cannot be attributed exclusively to explicit coordinate localization.

The other readout variants exhibited different failure patterns. Global max pooling produced high overall MAEs of 22.30 BPM on PURE and 22.20 BPM on UBFC-rPPG. This operation retained only the largest activation in each feature channel while discarding both its frequency position and the remaining activation distribution. Its poor overall performance therefore cannot be attributed solely to coordinate removal. The dense regression head produced MAEs of 10.06 BPM on PURE and 5.42 BPM on UBFC-rPPG. On PURE, most of the error arose from the same subject with high HR values, whereas on UBFC-rPPG, dense regression remained less accurate than soft-argmax. Although the flattened features retained their frequency-dependent ordering, the dense head estimated HR through unconstrained scalar regression rather than an explicit mapping to BPM coordinates. These results are therefore more consistent with limitations of unconstrained scalar regression, particularly under mismatch between the training and test HR ranges, than with a general failure to use the CWT representation.

Replacing CHROM with POS while retaining the frequency-preserving soft-argmax architecture changed the MAE by only 0.01 BPM on PURE and 0.15 BPM on UBFC-rPPG. The performance of the proposed architecture, therefore, did not depend strongly on the choice between these two color-projection front-ends.

The controlled ROI-fusion experiment similarly showed that additional architectural complexity was not beneficial in this setting. Cross-attention produced slightly higher pooled errors than element-wise averaging on both PURE and UBFC-rPPG, with no significant subject-level improvement. Only 3 of 10 PURE subjects and 14 of 38 UBFC-rPPG subjects improved, and no consistent advantage was observed across the PURE motion conditions. Moreover, the learned pairwise frequency-to-frequency attention distributions were close to uniform and showed only weak associations with the evaluated input-quality measures. Because the implemented module treated frequency bins rather than the three ROIs themselves as attention tokens, these findings should not be interpreted as a direct test of adaptive ROI-quality weighting. Instead, they indicate that the implemented cross-ROI frequency-attention formulation did not provide a measurable advantage over simple averaging. Future work should evaluate adaptive weighting or quality-aware gating based explicitly on ROI-specific signal quality; these mechanisms were not evaluated in the present study. Accordingly, simple element-wise averaging remains the primary fusion strategy in the current model, while quality-aware fusion is left as an important direction for future work.

Overall, the results support the complete soft-argmax readout formulation as a more reliable approach than the evaluated GAP-based readouts within the frequency-preserving architecture. The bounded controls show that output-range restriction alone was insufficient to reproduce soft-argmax performance on either primary dataset, although bounding contributed substantially on UBFC-rPPG. The additional Conv1D-removal ablation and parameter-count comparison further indicate that the advantage cannot be explained simply by the presence of the shared local Conv1D feature extractor or by greater model capacity. However, the present experiments do not provide a complete component-by-component decomposition of the structured readout. In particular, probabilistic normalization and coordinate-based expectation remain coupled, and we did not evaluate alternative normalization functions while retaining the same frequency-bin scores and expectation operator or a fully parameter-matched bounded scalar head designed specifically for this decomposition. The conclusion is therefore limited to the combined structured soft-argmax design and should not be interpreted as evidence that frequency-coordinate preservation alone is the causal factor or as evidence of general superiority over raw-video rPPG models or other estimator families.

The subject-macro and non-overlapping sensitivity analyses indicate that the main readout comparison was not an artifact of segment pooling or the 0.5 s stride. Equal weighting of subjects preserved the principal MAE ordering, and non-overlapping 10 s tilings reproduced the overlapping results with essentially unchanged method rankings. This is consistent with the subject-level statistical tests, which already treated subjects rather than overlapping segments as the independent units. Nevertheless, segment-pooled metrics remain useful as descriptive summaries of segment-wise behavior and are therefore retained alongside the subject-macro results.

### 5.2. How GAP and Soft-Argmax Use Frequency Information

CWT-based HR estimation differs structurally from conventional image classification. In an image-classification task, the presence of a feature is often more important than its precise spatial location, making spatially invariant aggregation desirable. In a CWT scalogram, however, the frequency axis has a direct physical interpretation: the frequency position of the cardiac ridge is related to HR. The way in which a model aggregates information along this axis can therefore affect its ability to estimate HR.

The frequency-axis sensitivity experiment further clarifies this point. Convolution across adjacent frequency bins should be interpreted as local feature integration, not as information leakage: neighboring CWT coefficients are deliberately combined to characterize the local shape and continuity of cardiac-frequency ridges. Frequency-axis downsampling, in contrast, reduces the sampling density of this coordinate. Strided decimation can, in principle, alias rapidly varying structure along the frequency axis if it is not preceded by explicit low-pass filtering, although the CWT scalograms used here are relatively smooth across neighboring frequencies and the present experiments do not demonstrate aliasing directly. The soft-argmax readout also returns a continuous expectation over BPM coordinates rather than quantizing the estimate to a single bin. This provides a plausible explanation for why reducing the axis from 300 to 100 coordinates produced little change, whereas the coarse 50-bin representation was less robust on PURE. The dataset-dependent 50-bin result prevents a general claim that finer resolution is always superior.

GAP averages the learned features over the frequency axis and consequently does not retain the frequency coordinate explicitly in the pooled representation. This does not mean that all position-related information is necessarily lost. The preceding convolutional layers may encode such information implicitly in their feature channels through learned filters, padding, and boundary effects. This interpretation is consistent with the matched backbone results: GAP produced reasonable estimates for many subjects but was less accurate than soft-argmax at the subject level on both PURE and UBFC-rPPG. GAP should therefore not be regarded as universally unsuitable for CWT-based HR estimation. Rather, it provides an indirect representation of frequency-related information that may be less reliable than an explicitly frequency-localizing readout.

GMP exhibited a different behavior from GAP. Although neither operation retains the frequency coordinate explicitly, global-max preserves only the strongest activation in each feature channel and discards the remaining activation distribution. Its poor overall performance on both datasets therefore cannot be explained by coordinate removal alone. The difference between GAP and GMP indicates that the type of information retained after pooling, rather than only whether the explicit coordinate is removed, is important for HR regression.

Soft-argmax uses a more structured readout. The network produces one score for each BPM bin, converts these scores into a probability distribution, and calculates the expected BPM value. When the distribution is sharply peaked, the estimate approaches the BPM coordinate of the highest-scoring bin. This formulation retains an explicit relationship between the learned score profile and the BPM axis. It is differentiable and can therefore be trained jointly with the convolutional backbone.

Soft-argmax also constrains the prediction to the predefined BPM range because its output is a convex combination of the BPM-bin coordinates. The bounded GAP control tested whether this range constraint alone explained the improvement. On PURE, bounded GAP produced an MAE of 58.26 BPM for the high-HR subject, compared with 9.12 BPM for soft-argmax. On UBFC-rPPG, the three-seed sensitivity analysis yielded pooled MAEs of 3.83 ± 0.14 BPM for soft-argmax and 4.35 ± 0.13 BPM for bounded GAP, with the lower MAE preserved in every seed, although subject-level significance was seed-dependent. Output bounding therefore contributed to performance but did not reproduce the complete soft-argmax behavior. Nevertheless, probabilistic normalization and coordinate-based expectation remain coupled, so no single soft-argmax component is established as the sole cause of the improvement.

The soft-argmax readout can be interpreted as a learned form of frequency localization. It is not mathematically identical to FFT-based peak selection because it operates on a learned BPM score profile rather than on a directly calculated Fourier spectrum. Nevertheless, both approaches estimate HR by identifying a position along a frequency-related axis. FFT-based estimators select a peak from a measured spectral profile, whereas soft-argmax calculates the expected coordinate of a learned probability distribution. The contribution of the proposed method is therefore not a new definition of spectral peak estimation, but the integration of a structured, differentiable frequency-localization readout into a trainable CWT-CNN.

The direct comparison with CWT-argmax further separates the value of explicit CWT frequency localization from the additional effect of learning the structured readout. On PURE, the two approaches had similar subject-level performance and no significant paired MAE difference, indicating that explicit localization of the CWT frequency structure accounted for much of the achievable accuracy in this dataset. On UBFC-rPPG, by contrast, soft-argmax significantly reduced subject-level MAE relative to CWT-argmax with a moderate rank-biserial effect size. The learned structured readout therefore provided an additional benefit on UBFC-rPPG, but this benefit was not uniform across datasets. This result reinforces the need to distinguish the contribution of the CWT representation itself from the contribution of the learned readout.

Overall, the matched backbone results support the complete soft-argmax formulation as a more reliable readout than GAP in the evaluated pipeline. The comparison with CWT-argmax shows that this conclusion should not be generalized to all forms of explicit frequency localization: the learned readout provided a significant additional benefit over non-learned CWT-argmax on UBFC-rPPG, but not on PURE. The experiments do not establish explicit coordinate preservation as the sole cause of the improvement, nor do they imply that GAP must fail in every CWT-based architecture. Instead, they show that directly linking the learned representation to the BPM coordinate provides a useful inductive structure relative to scalar GAP regression, while the incremental value of learning beyond direct CWT localization is dataset dependent.

### 5.3. Residual Errors and Controlled Condition Findings

The proposed architecture reduced error relative to the evaluated GAP-based readouts, but it did not eliminate errors associated with HR-range mismatch or degraded input signals. On PURE, all 582 segments with reference HR ≥ 120 BPM originated from sub07. When this subject was held out, the training subjects covered 44.0–103.8 BPM and contained no segments above 110 BPM, whereas the test subject covered 118.1–143.6 BPM. The soft-argmax model was substantially more robust than scalar regression in this out-of-range condition, but its error increased sharply at the extreme upper range. Importantly, in the 120–140 BPM interval, the classical FFT estimators achieved lower MAE than soft-argmax (4.11 BPM for CHROM-FFT and 4.00 BPM for POS-FFT versus 8.29 BPM), so the structured readout should not be interpreted as uniformly superior to classical frequency-domain estimation. The exploratory synthetic frequency-axis augmentation did not consistently improve soft-argmax across three seeds, although it reduced GAP error without producing any predictions within ±5 BPM. These results support insufficient training-range coverage as an important contributor to the high-HR failure, but they do not establish it as the sole cause.

On UBFC-rPPG, the three largest-error subjects did not support a single physical explanation. Independent contact-PPG analysis confirmed that the reference HR was valid, while the camera-derived signals contained dominant non-cardiac components. Subject26 showed a projection-sensitive pattern because the green channel recovered a frequency closer to the reference than CHROM or POS. Subject25 showed a projection-persistent RGB-domain artifact despite little rigid ROI motion, whereas subject27 showed a motion-associated pattern. Bounded GAP also produced large errors for all three subjects and was more accurate than soft-argmax for two of them. Thus, changing between the two bounded readouts did not consistently resolve these cases. The observations refine the earlier shared-failure interpretation: the largest errors arose upstream of the final readout, but the upstream pathway differed across subjects. The physical sources of the subject25 and subject26 patterns could not be determined from the available measurements.

The exclusion-sensitivity analysis further showed that the post hoc reference-quality screening influenced absolute UBFC-rPPG error levels but not the relative readout conclusion. The four excluded subjects exhibited markedly atypical reference-HR variability and extreme reference values. When they were restored to the LOSO evaluation, MAE increased for both soft-argmax and matched GAP, while soft-argmax remained significantly better at the subject level. This supports reporting the 38-subject cohort as the primary analysis while making clear that the exclusion improves absolute benchmark values and should not be interpreted as inconsequential.

The reference-free quality score partially addressed the practical limitation of the reference-dependent non-cardiac dominance measure. After correcting the procedure so that feature standardization and the rejection threshold were calibrated exclusively from training subjects within each LOSO fold, 15% rejection reduced pooled UBFC-rPPG MAE from 3.82 to 3.13 BPM, essentially unchanged from the previously obtained 3.14 BPM. This indicates that the original calibration leakage had negligible numerical impact on the reported result. Nevertheless, the weak segment-level association between Q and estimation error, the early plateau of the risk-coverage behavior, and the inability to detect sharp but incorrect ridges show that input-only rejection cannot fully solve the problem. Moreover, the feature combination and 15% rejection proportion were selected post hoc on UBFC-rPPG and have not been validated on an independent dataset. The quality score should therefore be regarded as exploratory rather than as a deployment-ready quality gate.

The input-level analyses provide a signal-processing interpretation of this illumination effect. From high to low illumination, cardiac-band relative energy decreased from 0.202 to 0.173 and peak prominence decreased from 1.51 to 1.32, while the absolute deviation of the dominant CWT peak from the reference HR increased from 7.04 to 11.16 BPM. These changes indicate that reduced illumination weakens and disperses the cardiac-frequency structure before the readout stage. In the FFT-based estimators, the entire 10 s window is summarized by a single global spectrum, so transient illumination fluctuations, motion, or locally degraded intervals contribute to the same spectral estimate and can obscure the cardiac peak. The CWT, by contrast, retains temporal localization of frequency content, which may help persistent cardiac-related structure remain distinguishable from transient or broadband disturbances even when its amplitude is reduced. This interpretation does not imply intrinsic noise suppression or universally superior spectral resolution by the CWT; rather, the observed pattern is consistent with a contribution from the retention of time-localized frequency structure. The similar degradation of the learned model and CWT-argmax further suggests that this behavior is associated mainly with properties shared by the CWT representation and frequency localization rather than with the soft-argmax readout alone.

The BH-rPPG analysis provided a complementary evaluation of illumination. Under LOSO cross-validation, error increased as illumination decreased for all evaluated methods. The proposed method showed significantly less subject-level degradation from high to low illumination than CHROM-FFT and POS-FFT after Holm’s correction. However, no significant difference was detected between the proposed method and the non-learned CWT-argmax baseline. This result indicates that the proposed method was less affected by illumination reduction than the direct FFT-based estimators, but it does not establish an additional illumination-robustness benefit from the learned soft-argmax readout over CWT-argmax. Instead, the pattern is consistent with the robustness difference being mainly associated with CWT-based frequency localization.

Cross-dataset transfer was asymmetric. Training on PURE and testing on UBFC-rPPG produced an MAE of 4.08 BPM, whereas training on UBFC-rPPG and testing on PURE produced an MAE of 8.44 BPM. The UBFC-rPPG training pipeline already restricted reference HR to 50–130 BPM, and the retained cohort actually covered 58.3–124.6 BPM. In the reverse transfer direction, PURE segments within this observed range had an MAE of 2.61 BPM, whereas segments below and above the range had MAEs of 16.72 and 7.88 BPM, respectively. The mismatch was therefore concentrated particularly on the low-HR side, where 38.2% of PURE test segments fell below the empirically observed UBFC-rPPG training range; an additional 8.2% fell above it. This post hoc decomposition supports HR-range mismatch as an important contributor to the transfer asymmetry but not as its sole explanation. Differences in illumination, camera characteristics, motion patterns, subject populations, and other dataset-specific factors remain potential confounders.

The agreement analysis showed only small mean bias on both primary datasets. The identified high-error cases mainly widened the observed limits of agreement rather than producing a consistent model-wide overestimation or underestimation. Thus, the residual errors were concentrated in specific HR-range and input-quality conditions rather than reflecting a uniform directional bias.

### 5.4. Scope and Relation to Modern rPPG Models

This study examines frequency preservation and readout design within a fixed CHROM-CWT pipeline rather than competing with state-of-the-art raw-video rPPG models. Modern methods such as DeepPhys, TS-CAN, PhysNet, EfficientPhys, and PhysFormer use different input representations, preprocessing procedures, supervision targets, and spatiotemporal architectures. Their published results are therefore not directly comparable with the controlled experiments reported here.

The matched backbone GAP variant provides the most direct architectural comparison because only the readout differs. The conventional GAP-based CWT-CNN is retained as a broader baseline using the same CHROM-CWT inputs but a different backbone. Among the FFT-based estimators, CHROM-FFT provides the matched transform comparison because it uses the same CHROM projection and segmented input signal as the proposed pipeline, with the principal change being CWT versus FFT-based spectral estimation. POS-FFT is retained as an additional classical reference rather than a matched ablation because it changes the color-projection method and projection-window configuration. A unified comparison with modern raw-video and time–frequency models would be valuable future work, but it addresses a broader question than the architectural analysis conducted in this study.

### 5.5. Limitations and Future Work

This study provided evidence supporting a structured frequency-localizing readout within a controlled CHROM-CWT pipeline, while also identifying several directions for further validation and extension.

First, the primary evaluation on PURE and UBFC-rPPG demonstrated consistent subject-level benefits of the proposed readout under subject-independent validation. BH-rPPG further showed that CWT-based frequency localization was less affected by illumination reduction than the evaluated FFT-based estimators. These datasets provided controlled evaluations of readout design, motion, and illumination, but broader validation is needed across more diverse skin tones, cameras, compression settings, head poses, occlusions, and naturalistic motion. Future studies should therefore evaluate the method on larger and more heterogeneous datasets and under less constrained acquisition conditions.

A further limitation is that the primary LOSO results were obtained from one training initialization per fold, and the TensorFlow global random seed was not fixed in the original primary training scripts. Although the added three-seed UBFC-rPPG sensitivity analysis preserved the MAE-based ordering between soft-argmax and bounded GAP, the effect magnitude and subject-level statistical significance were seed-dependent. Future evaluations should repeat the complete LOSO procedure across multiple explicitly controlled initializations for all principal learned readouts.

Second, the input-signal analyses showed that the proposed readout reduced errors arising from scalar regression but could not recover cardiac information that was already weak or distorted in the camera-derived signal. The projection-sensitive, projection-persistent, and motion-associated patterns identified in UBFC-rPPG provide concrete examples of distinct upstream failure modes. Because each pattern was represented by a single subject, future work should test whether these categories generalize across larger cohorts and should incorporate more detailed measurements of local facial deformation, illumination variation, and skin reflectance. The exploratory scalogram-quality score showed that reference-free input characteristics could identify some unreliable segments, and fold-wise train-only calibration removed held-out-subject leakage without materially changing the result. However, the feature definition and rejection proportion remain post hoc choices and have not been validated externally. A practical quality-control mechanism will therefore require prespecified train-only calibration, out-of-fold evaluation, subject-wise coverage analysis, and independent external validation.

Third, the high-HR PURE subject provided a clear example of mismatch between the observed training and test HR ranges: its LOSO training fold covered 44.0–103.8 BPM, whereas the held-out subject covered 118.1–143.6 BPM. In addition, the proposed soft-argmax output is bounded by construction to 45–150 BPM; therefore, the highest observed PURE values approach the structural upper limit of the readout and should not be interpreted as an unconstrained test of extrapolation beyond 150 BPM. The structured readout was substantially more robust than scalar regression under this mismatch, but only one PURE subject exhibited this extreme upper-range condition, and UBFC-rPPG also contained relatively few segments above 120 BPM. Conventional oversampling was impossible for the critical PURE fold because no genuine high-HR training segments were available, and exploratory frequency-axis augmentation did not consistently improve soft-argmax across three seeds. Future work should therefore evaluate the method using datasets with broader and more balanced genuine HR coverage and should compare oversampling, loss weighting, and HR-targeted augmentation when representative samples are actually available. Such augmentation should preserve realistic physiological and image-domain characteristics.

Fourth, the bounded GAP control showed that output-range restriction alone was insufficient to reproduce the soft-argmax result. The additional inverse ablation further showed that removing the shared post-fusion Conv1D block did not change the relative ordering of GAP, bounded GAP, and soft-argmax on PURE, and the proposed model used slightly fewer trainable parameters than the matched GAP controls. These results make local Conv1D processing and model capacity less likely explanations for the observed advantage. However, probabilistic normalization and coordinate-based expectation remain coupled in the proposed readout. We did not evaluate alternative normalization functions while retaining identical frequency-bin scores and the coordinate-expectation operator, nor did we construct a fully parameter-matched bounded scalar head specifically for a progressive component-level decomposition. Future ablations should isolate these remaining components to clarify which elements of the structured readout provide the greatest benefit and whether a simpler architecture could achieve comparable performance.

The frequency-resolution analysis also showed that the full 300-bin axis is not a strict requirement: 100-bin variants were statistically comparable on both primary datasets, while the 50-bin effect was dataset-dependent. Because the downsampled variants used strided convolution without a dedicated anti-aliasing filter, this experiment does not establish the optimal frequency sampling density or isolate potential aliasing effects. Future work should compare alternative frequency grids and explicitly anti-aliased downsampling schemes.

The cross-attention control did not improve performance consistently across subjects or motion conditions, and its pairwise frequency-to-frequency attention distributions remained close to uniform and showed only weak associations with the evaluated input-quality measures. Because frequency bins rather than ROIs themselves were used as attention tokens, this negative result is specific to the implemented cross-ROI frequency-attention formulation and should not be interpreted as evidence against adaptive ROI weighting in general. ROI-quality supervision, signal-quality-based adaptive weighting, explicit signal-to-noise targets, or gating mechanisms that directly suppress degraded ROIs were not evaluated in the present study and should be examined in future work using independently defined ROI-quality criteria.

Fifth, the present study focused on an interpretable hybrid pipeline rather than direct comparison with state-of-the-art raw-video models. A future unified evaluation using a common framework such as rPPG-Toolbox would enable matched comparisons with recent raw-video and time–frequency models under identical subject splits, preprocessing, and metrics. Such a comparison would position the proposed frequency-aware readout relative to higher-capacity end-to-end approaches while preserving the controlled architectural analysis conducted here.

Sixth, the primary descriptive metrics were originally calculated from highly overlapping 10 s segments. The added subject-macro and non-overlapping sensitivity analyses showed that the principal method rankings were preserved when each subject was weighted equally and when independent 10 s test windows were used. However, the segment-level Bland–Altman analysis remains a descriptive view of the observed error distribution because overlapping windows and repeated observations violate the assumptions of conventional independent-sample agreement analysis. Future work should apply repeated-measures or subject-level Bland–Altman methods for formal agreement inference.

Seventh, the 15 BPM UBFC-rPPG reference-variability threshold was selected post hoc after inspection of the reference data. Although the criterion was independent of camera-derived signals and model predictions, it affected the reported absolute error values. The added 42-subject sensitivity analysis showed that including the four excluded subjects increased MAE for both matched readouts while preserving the relative ordering and significant subject-level advantage of soft-argmax. Future benchmarking should prespecify reference-quality criteria where possible and report exclusion-sensitivity results when post hoc screening is unavoidable.

Finally, the measured post-ROI processing cost was compatible with the 0.5 s update interval on the evaluated laptop CPU, but video acquisition and face-landmark detection were not included in the timing analysis; fully end-to-end real-time performance therefore remains to be evaluated. The present findings support further development of CWT-based rPPG systems for practical monitoring, particularly under conditions in which signal quality varies. Translation to real-world use will require privacy-preserving handling of facial and physiological data, uncertainty estimation, automated rejection of unreliable segments, and external validation before use in diagnosis, emergency monitoring, or clinical decision-making.

## 6. Conclusions

This study investigated how readout design and input signal quality affect HR estimation from CWT scalograms. Within a controlled CHROM-CWT pipeline, the proposed frequency-preserving CNN with a soft-argmax readout achieved lower error than the matched backbone GAP variant. Additional frequency-resolution experiments showed that the original 300-bin sampling density was not essential: reducing the retained axis to 100 bins produced statistically comparable performance, whereas the 50-bin effect was dataset-dependent. Thus, the results suggest that retaining an explicit frequency-coordinate representation through the readout is more important than preserving all 300 sampled coordinates. Subject-macro averaging and non-overlapping-window sensitivity analyses preserved the principal method rankings, indicating that the main readout comparison was not dependent on unequal subject weighting or heavy overlap between adjacent test segments. The UBFC-rPPG exclusion-sensitivity analysis likewise showed that restoring the four post hoc reference-quality exclusions increased absolute MAE for both matched readouts but did not change their relative ordering or the subject-level conclusion.

The residual analyses showed that the readout could not resolve all failures originating from degraded camera-derived signals. On BH-rPPG, reduced illumination weakened the cardiac-related component in the CHROM signal and CWT representation: cardiac-band relative energy and peak prominence decreased, while peak deviation from the reference HR increased. Nevertheless, the proposed model achieved the lowest segment-pooled MAE under low illumination, and the CWT-based estimators showed smaller high-to-low degradation than the evaluated FFT-based estimators. This pattern is consistent with a benefit from retaining time-localized frequency structure, rather than with intrinsic noise suppression by the CWT.

The bounded-GAP control showed that restricting predictions to the same BPM range as soft-argmax was insufficient to reproduce the full performance advantage of soft-argmax. Additional ablation showed that the advantage was not explained by the shared local Conv1D feature extractor or by a larger parameter count: removing the shared Conv1D block did not change the relative ordering of the readouts on PURE, and the proposed model contained 119,921 trainable parameters compared with 122,001 for the matched GAP controls. Because probabilistic normalization and coordinate-based expectation remain coupled, and alternative normalization schemes or a fully parameter-matched bounded scalar head were not evaluated, the benefit should be attributed to the structured soft-argmax formulation as a whole rather than to explicit frequency-coordinate localization alone.

Overall, the findings support the structured soft-argmax readout as an effective approach for CWT-based HR estimation relative to the evaluated GAP-based readouts. However, because probabilistic normalization and coordinate-based expectation remain coupled, the observed advantage should be attributed to the structured soft-argmax formulation as a whole rather than to frequency-coordinate preservation alone. The findings also indicate that reliable CWT-based rPPG requires both an appropriate readout design and explicit management of errors caused by weak input signals, motion, spectral ambiguity, and mismatch between the training and application HR ranges.

## Figures and Tables

**Table 1:** Primary within-dataset HR estimation results under LOSO cross-validation.

Dataset	Method	MAE	RMSE	r	Within ±5 BPM
UBFC-rPPG	CHROM-FFT	5.68	10.33	0.83	72.2%
	POS-FFT	4.79	7.82	0.89	73.7%
	CWT-argmax	5.58	11.80	0.78	77.5%
	Proposed	**3.82**	**6.70**	**0.91**	**79.7%**
PURE	CHROM-FFT	3.10	5.69	0.97	84.6%
	POS-FFT	3.02	**5.43**	**0.97**	85.1%
	CWT-argmax	3.10	8.89	0.92	**89.2%**
	Proposed	**2.98**	6.00	0.97	87.1%

**Table 2:** Matched backbone readout comparison under LOSO cross-validation. All variants use the same CHROM-CWT input, frequency-preserving backbone, ROI fusion, and training protocol. Only the readout head differs.

Dataset	Readout	MAE	RMSE	r	Within ±5 BPM
UBFC-rPPG	GAP	5.14	7.38	0.89	64.7%
	GMP	22.20	24.03	0.81	3.5%
	Dense	5.42	7.61	0.88	60.1%
	Soft-argmax (proposed)	**3.82**	**6.70**	**0.91**	**79.7%**
PURE	GAP	8.09	16.88	0.78	72.0%
	GMP	22.30	30.59	0.41	1.8%
	Dense	10.06	17.32	0.73	63.4%
	Soft-argmax (proposed)	**2.98**	**6.00**	**0.97**	**87.1%**

**Table 3:** Bounded output control under LOSO cross-validation. All variants use the same frequency-preserving backbone, and values are segment-pooled MAE (BPM). The two PURE subset rows are post hoc diagnostic analyses; the all-subject rows are the primary comparisons.

Dataset	Evaluation Subset	Unbounded GAP	Bounded GAP	Soft-Argmax
UBFC-rPPG	All subjects (*n* = 38)	5.14	4.19	3.82
PURE	All subjects (*n* = 10)	8.09	8.53	2.98
PURE	Typical-range subjects (*n* = 9)	3.36	2.93	2.29
PURE	High HR subject (*n* = 1)	50.12	58.26	9.12

**Table 4:** Comparison of CHROM and POS-derived inputs under the proposed frequency-preserving soft-argmax architecture.

Input Signal	PURE MAE	UBFC-rPPG MAE
CHROM-derived signal	**2.98**	**3.82**
POS-derived signal	2.99	3.97

**Table 5:** Diagnostic results for the PURE motion conditions under LOSO cross-validation after post hoc exclusion of the high HR subject examined in Section 4.3.1. The primary within-dataset results include all subjects. (N) denotes the number of analyzed recording sessions.

Motion Condition	N (Sessions)	MAE	RMSE	r	Within ±5 BPM
Steady	9	1.99	2.52	0.97	96.0%
Talking	8	3.50	4.43	0.96	73.3%
Slow Translation	9	2.11	2.73	0.97	93.7%
Fast Translation	9	2.32	2.92	0.96	91.1%
Small Rotation	9	1.87	2.44	0.97	96.2%
Medium Rotation	9	2.22	2.77	0.97	92.7%
All Analyzed Sessions	53	2.29	3.02	0.96	91.9%

**Table 6:** BH-rPPG results under 35-subject LOSO cross-validation. Segment-pooled MAEs were calculated over 8473 held-out test segments. CWT-argmax uses the same CHROM-CWT scalograms as the proposed model but estimates HR through non-learned temporal averaging and frequency-bin selection. The final column reports the segment-pooled low-to-high illumination difference. Statistical comparisons were based on subject-level degradation values and are reported in the text.

Method	Low MAE	Medium MAE	High MAE	Overall MAE	Pooled Low–High Difference
CHROM-FFT	13.81	9.15	7.12	10.05	6.69
POS-FFT	12.39	**5.64**	4.54	7.55	7.86
CWT-argmax	11.17	7.77	7.09	8.69	4.08
Proposed	**9.30**	7.02	5.85	**7.41**	**3.45**

**Table 7:** Cross-dataset transfer without fine-tuning. The all-segment MAE is the primary result. Restricted-subset MAEs are reported only as post hoc diagnostic decompositions. For UBFC-rPPG-to-PURE transfer, the reported subset corresponds to PURE segments within the empirically observed UBFC-rPPG training range of 58.3–124.6 BPM; segments below and above this range had MAEs of 16.72 and 7.88 BPM, respectively.

Dataset Used for Training	Dataset Used for Testing	No. of Test Segments	Overall MAE	Overall Pearson’s *r*	Restricted Subset MAE
PURE	UBFC-rPPG	3386	4.08	0.92	3.06
UBFC-rPPG	PURE	5890	8.44	0.90	2.61 (within 58.3–124.6 BPM)

## Data Availability

The PURE, UBFC-rPPG, and BH-rPPG datasets analyzed in this study are publicly available from their respective providers, subject to the applicable terms of use. The processed results and implementation code supporting the findings of this study are available from the corresponding author upon reasonable request.

## Appendix A. HR-Stratified Performance

Table A1 and Table A2 report the complete HR-stratified evaluation used in the HR-range analysis. Bands were defined from the reference HR, and all metrics were calculated from the existing held-out LOSO predictions without retraining or re-inference.

**Table A1:** HR-stratified performance on PURE. HR bands were defined using reference HR. Metrics were calculated from held-out LOSO test predictions.

HR Band (BPM)	*n*	Method	MAE	RMSE	Within ±5 BPM
<60	2363	CHROM-FFT	2.52	3.51	89.8%
<60	2363	POS-FFT	2.64	4.07	89.3%
<60	2363	CWT-argmax	1.85	2.39	95.1%
<60	2363	Matched GAP	3.08	4.23	82.1%
<60	2363	Bounded GAP	2.14	2.70	93.4%
<60	2363	Proposed soft-argmax	2.14	2.69	93.5%
60–80	2615	CHROM-FFT	2.93	3.83	83.7%
60–80	2615	POS-FFT	2.86	3.71	84.4%
60–80	2615	CWT-argmax	2.16	2.94	91.5%
60–80	2615	Matched GAP	2.70	3.54	86.6%
60–80	2615	Bounded GAP	2.65	3.32	88.8%
60–80	2615	Proposed soft-argmax	2.09	2.83	92.2%
80–100	236	CHROM-FFT	7.26	9.64	40.7%
80–100	236	POS-FFT	5.85	7.10	47.9%
80–100	236	CWT-argmax	6.94	8.12	35.6%
80–100	236	Matched GAP	10.56	11.21	8.1%
80–100	236	Bounded GAP	8.89	9.42	9.7%
80–100	236	Proposed soft-argmax	5.47	6.54	47.9%
100–120	94	CHROM-FFT	3.68	6.45	83.0%
100–120	94	POS-FFT	3.51	6.10	83.0%
100–120	94	CWT-argmax	3.87	6.05	75.5%
100–120	94	Matched GAP	15.28	19.03	18.1%
100–120	94	Bounded GAP	20.66	22.34	4.3%
100–120	94	Proposed soft-argmax	3.49	5.14	78.7%
120–140	554	CHROM-FFT	4.11	12.04	86.1%
120–140	554	POS-FFT	4.00	11.82	86.5%
120–140	554	CWT-argmax	8.84	23.56	83.2%
120–140	554	Matched GAP	49.60	50.43	0.0%
120–140	554	Bounded GAP	58.08	58.71	0.0%
120–140	554	Proposed soft-argmax	8.29	15.55	58.5%
≥140	28	CHROM-FFT	10.35	24.58	78.6%
≥140	28	POS-FFT	5.28	14.45	85.7%
≥140	28	CWT-argmax	49.08	60.64	0.0%
≥140	28	Matched GAP	68.14	68.32	0.0%
≥140	28	Bounded GAP	72.76	72.82	0.0%
≥140	28	Proposed soft-argmax	29.19	31.13	0.0%

**Table A2:** HR-stratified performance on UBFC-rPPG. HR bands were defined using reference HR. Metrics were calculated from held-out LOSO test predictions.

HR Band (BPM)	*n*	Method	MAE	RMSE	Within ±5 BPM
<60	44	CHROM-FFT	3.89	5.23	56.8%
<60	44	POS-FFT	4.03	5.47	56.8%
<60	44	CWT-argmax	3.89	4.24	81.8%
<60	44	Matched GAP	8.60	9.20	4.5%
<60	44	Bounded GAP	4.48	5.04	75.0%
<60	44	Proposed soft-argmax	8.17	12.29	52.3%
60–80	494	CHROM-FFT	7.56	10.71	52.2%
60–80	494	POS-FFT	7.52	10.68	52.6%
60–80	494	CWT-argmax	6.29	9.67	59.5%
60–80	494	Matched GAP	8.58	11.29	34.2%
60–80	494	Bounded GAP	6.64	9.20	54.0%
60–80	494	Proposed soft-argmax	6.94	10.47	58.1%
80–100	1282	CHROM-FFT	6.47	11.56	73.9%
80–100	1282	POS-FFT	5.09	8.40	76.4%
80–100	1282	CWT-argmax	6.46	12.60	76.5%
80–100	1282	Matched GAP	4.21	6.69	76.6%
80–100	1282	Bounded GAP	4.40	6.71	70.8%
80–100	1282	Proposed soft-argmax	3.84	6.85	81.3%
100–120	1458	CHROM-FFT	4.60	9.35	77.0%
100–120	1458	POS-FFT	3.79	6.29	78.3%
100–120	1458	CWT-argmax	4.58	11.43	83.3%
100–120	1458	Matched GAP	4.55	5.96	68.1%
100–120	1458	Bounded GAP	3.14	4.41	80.0%
100–120	1458	Proposed soft-argmax	2.72	4.46	85.8%
120–140	108	CHROM-FFT	3.03	6.77	84.3%
120–140	108	POS-FFT	2.56	3.56	84.3%
120–140	108	CWT-argmax	6.17	17.24	89.8%
120–140	108	Matched GAP	7.16	9.06	40.7%
120–140	108	Bounded GAP	4.52	5.68	65.7%
120–140	108	Proposed soft-argmax	2.38	3.66	88.0%
